# Amino-isocyanoacridines: Novel, Tunable Solvatochromic Fluorophores as Physiological pH Probes

**DOI:** 10.1038/s41598-019-44760-9

**Published:** 2019-06-03

**Authors:** Miklós Nagy, Dávid Rácz, Zsolt László Nagy, Péter Pál Fehér, Sándor Lajos Kovács, Csaba Bankó, Zsolt Bacsó, Alexandra Kiss, Miklós Zsuga, Sándor Kéki

**Affiliations:** 1 Department of Applied Chemistry, University of Debrecen, 4010 Debrecen, Hungary; 2 Research Centre for Natural Sciences, Hungarian Academy of Sciences, H-1117 Budapest, Magyar tudósok körútja 2, Budapest, Hungary; 3 University of Debrecen, Medical and Health Science Center, Faculty of Medicine, Department of Biophysics and Cell Biology, 4010 Debrecen, Hungary; 4 Department of Biotechnology and Microbiology, Faculty of Science, University of Debrecen, Debrecen, 4010 Hungary

**Keywords:** Fluorescent probes, Single-molecule fluorescence

## Abstract

Amino-isocyanoacridines (**ICAAc**s), as first members of their class, turned out to be a novel, multifunctional acridine orange (AO) type dye family with a number of additional favorable properties. They have enhanced solvatochromic emission range, low quantum yields (Φ_F_ = 2.9–0.4%) in water, reduced basicity (pK_a_ = 7.05–7.58), and their optical behavior could be fine-tuned by complexation with Ag(I) ions, too. Based on both their vibronic absorption and the charge transfer bands, **ICAAc**s can be applied as stable pH-probes with great precision (2–3% error) in the physiological pH range of 6–8 using UV-vis and fluorescence detection. The dyes are also able to sense pH change in different microenvironments, such as the Stern layer, as it was demonstrated on sodium lauryl sulfate micelles. The optical behavior of the **ICAAc** derivatives is discussed based on high-level quantum chemical calculations. All three dyes are well-applicable with conventional epifluorescence imaging. Furthermore, at the blue excitation, **diMICAAc** is optimally suited as a whole-cell probe for both the conventional microscopic and the laser-illumination studies, like flow- and imaging cytometric, or confocal laser-scanning microscopic examinations.

## Introduction

Since the first report of 3,6-bis(dimethylamino)acridine or acridine orange (AO) as a fluorochromatic stain^1^, acridinediamines have become one of the most investigated class of fluorescent dyes owing to their multifunctional properties. It exhibits affinity for nucleic acids and can be used to fluorometrically differentiate between DNA and RNA^2–4^. Moreover, AO shows marked accumulation in the acidic lysosomes of cancer cells, shows stronger cytotoxic effects under blue light or UV-C illumination making it a promising candidate for effective anti-cancer therapy^5,6^. As a weak basic dye (pK_a_ = 10.5) AO is used as an intracellular pH indicator due to its low molecular weight and hydrophobic nature. Precise measurement of intracellular pH can provide critical information for studying physiological and pathological processes, such as cell death, cancers, and cell proliferation^7^. For quantitative pH measurements, the use of luminescent sensors is desired^8,9^. The most commonly used cellular pH-sensitive stains are BCECF^10–12^, carboxySNARF-1^13,14^, BODIPYs^15^ and 8-Hydroxypyrene-1,3,6-trisulfonic acid (HPTS)^16–19^. Because of their cheapness and easy preparation fluorescein and fluorescein derivatives^20,21^ are also still used. Although being excellent physiological pH probes, a few of the dyes listed above have serious drawbacks such as cell impermeability, leakage through the membranes, suboptimal properties in terms of photostabilities or quantum yields, complex structure, therefore complicated synthesis and high price. In addition to pH-sensing, AO is extensively used for the mapping of microenvironments, such as the interior or Stern layer of reverse or normal micelles and water in oil microemulsions^22–27^. Moreover, very recently Yeasmin *et al*. showed^28^ by utilizing a spiro-rhodamine pH-probe, which selectively interacted with the anionic interfacial Stern layer, that it is possible that a defined curvature at the membrane interface controls its pH/polarity to exhibit specific bioactivity. We assume that an AO like smart dye would also be able to detect pH deviation in artificial and/or natural membranes.

A possible solution to prepare a “smarter” fluorescent dye could be the exchange of one of the dimethylamino groups of AO to a multifunctional electron withdrawing group, such as isocyanide. Despite the versatile chemistry of isonitriles, the field of isocyanoacridines remained completely unexplored until now. The incorporation of the isocyano group has many advantages: it can lower the pK_a_ of AO from 10.5 to around 7.0 yielding a powerful physiological pH probe, the reactive C-N bond can serve as a versatile base for many organic reactions, such as the Ugi reaction^29^, isonitriles easily form complexes with transition metal ions^30^ and the related isonitrile complexes of silver(I) are much less studied, in spite of interesting properties such as liquid crystalline behaviour^31,32^.

**Figure 1:**
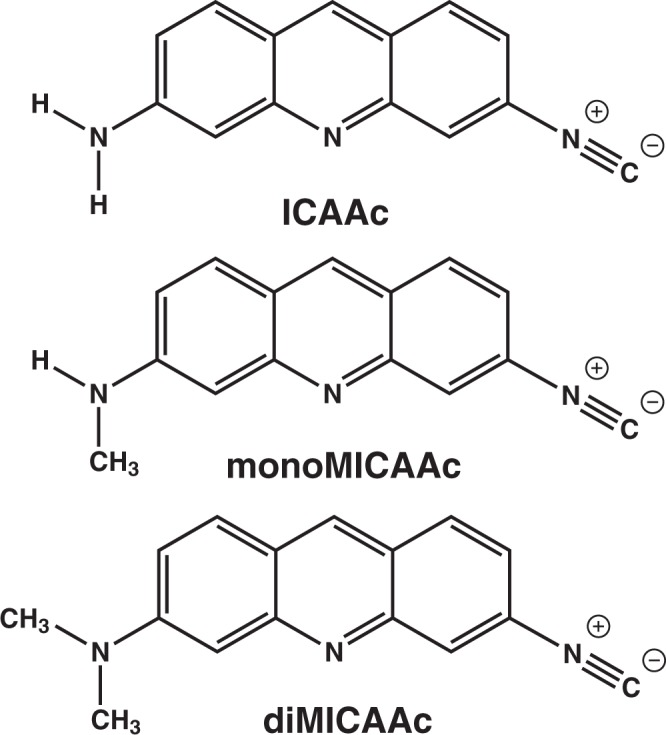
The structures and names of the dyes used in this study. 3-amino-6-isocyanoacridine (**ICAAc**), 3-N-methylamino-6-isocyanoacridine (**monoMICAAc**), 3-N,N-dimethylamino-6-isocyanoacridine (**diMICAAc**).

Recently, we developed a novel amino-isocyanonaphthalene (ICAN) based solvatochromic fluorophore family^33,34^. We assumed that by combining the best properties of our ICAN derivatives with those of AO we can obtain a novel dye family, amino-isocyanoacridines (**ICAAc**s) with significantly extended multifunctionality. 3-amino-6-isocyanoacridine (**ICAAc**) can be obtained by the treatment of proflavine with dichlorocarbene resulting the conversion of one of the amino groups to isonitrile (Fig. 1).

Hereby we report the development of a multifunctional isocyanoacridine based solvatochromic family with tunable properties. The photophysical behavior of the dyes, their applicability as physiological pH probes, their behavior in the micellar environment, their complexation with Ag(I) were investigated in detail and was backed by quantum chemical calculations. In addition, the cell staining ability of the **ICAAc** derivatives was also studied.

## Results and Discussion

### Steady-state fluorescence properties

All the quantum chemical (DFT) calculations, steady-state emission, absorption and excitation spectra in various solvents are presented in the Supporting Information (SI) for all the substances as separate chapters.

**Figure 2:**
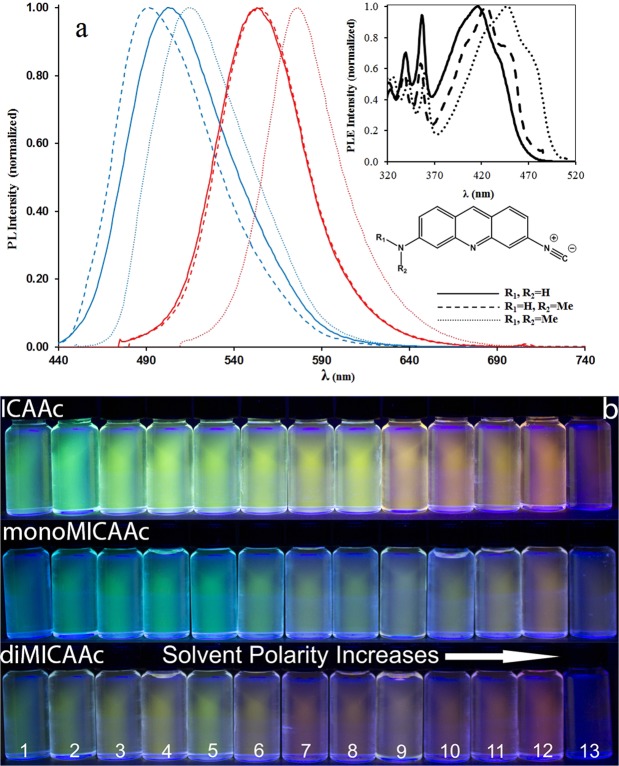
(**a**) The normalized emission spectra of the isocyanoacridine dyes recorded in hexane (blue) and water (red). The inset shows the excitation spectra recorded only in hexane. (**b**) Demonstration of the fluorescence properties of the **ICAAc** derivatives in different solvents (λ_ex_ = 365 nm). Solvents from left to the right are hexane (1), toluene (2), 1,4-dioxane (3), CH_2_Cl_2_ (4), CHCl_3_ (5), THF (6), MeCN (7), acetone (8), pyridine (9), methanol (10), DMF (11), DMSO (12), water (13).

The UV-vis and fluorescence properties of **ICAAc**, **monoMICAAc**, and **diMICAAc** for solvents of different polarity are summarized in Fig. 2a and Table 1. Independently of solvent polarity, similarly for all three compounds, a broad long wavelength absorption band is seen in the range of ~370–540 nm (Fig. 2a inset), which can be attributed to the internal charge transfer transition (ICT) between the donor amino and the acceptor isocyano groups. This ICT band is accompanied by overlapping structured absorption bands belonging to the locally excited state (LE) characteristic for the aromatic acridine ring in the region of ~300–370 nm. These structured bands are also present in the UV-vis spectrum of 2-aminoacridine^35^, however they are completely missing from the spectrum of both proflavine and acridine orange. The reappearance of the structured bands may be explained by the presence of the rigid electron withdrawing isocyanide group, which breaks the symmetry of the molecule compared to AO. While the position of the LE bands remains unaffected by changing the solvent polarity, the longer wavelength (ICT) absorption bands show a considerable redshift with increasing solvent polarity. Increasing the electron donating ability of the amino group by methylation also results in a bathochromic shift of the higher wavelength absorption bands. These shifts are greater than those observed when changing solvent polarity. That is, the higher the electron donating ability of the amino group the higher wavelength the ICT absorption peak is located at. As it is evident from Fig. 2 and Table 1, all three dyes have a considerable emission range spanning from green to orange. The emission maximum of **diMICAAc** in water is the highest of the three dyes with λ_em_ = 576 nm, which is 50 nm higher than that of AO (λ_em_ = 526 nm). In addition, AO has a solvatochromic range of approximately 30 nm, while in the case of **monoMICAAc** and **diMICAAc** this value is doubled to more than 60 nm.

**Table 1:** Comparison of the optical properties of the dyes in solvents of different polarity.

	Hexane (Polarity index = 0.0)			Dioxane (Polarity index = 4.8)			Water (Polarity index = 9.0)						μ_e_ − μ_g_/D (DFT calculation)	μ_e_ − μ_g_/D (Lippert Mataga)
	λ_em,max_ (nm)	λ_ex,max_ (nm)	Δν (cm^−1^)	λ_em,max_ (nm)	λ_ex,max_ (nm)	Δν (cm^−1^)	λ_em,max_ (nm)	λ_ex,max_ (nm)	Δν (cm^−1^)	ε (M^−1^cm^−1^)	Φ_F_ (%)	Δλ_em_ (nm)		
AO^*^	498	412	4191	500	426	3474	526	492	1313	44000	15	28	2^a^	2.27^a^
**ICAAc**	503	418	4158	515	431	3784	553	466	3376	10132	1.8	53	6.1	5.6 ± 1.4
**monoMICAAc**	491	425	3163	515	437	3466	554	472	3136	5196	2.9	63	6.8	8.1 ± 0.8
**diMICAAc**	514	447	2916	539	456	3377	576	497	2760	13344	0.4	62	7.9	8.9 ± 1.1

Emission (λ_em,max_), excitation (λ_ex,max_) maxima, Stokes shift (**Δν**), molar absorbance (**ε**) and quantum yield (**Φ**_F_), as well as theoretically calculated and experimentally estimated dipole moment difference data. ^a^Taken from ref.^38^. ^*^The optical data of AO was obtained from ref.^39^.

To get a better understanding of the solvatochromic behavior of the **ICAAc** derivatives Lippert-Mataga plots (Figs S14, 15) were constructed based on Eqs S1, S2 in the SI. As solvent polarity increases the emission maximum shifts bathochromically (Fig. S14b), due to the solvent reorientation around the excited dye, reducing the energy gap between the ground and excited state and as a result the energy of the emitted photon also decreases. Additionally, the quantum yields (Φ_F_) show a linear decrease with increasing solvent polarity parameter E_T_(30) values, which is in line with the data of Fig. 2 and Table 1. All our dyes have very low quantum yields (Φ_F_ = 2.9–0.4%) in water, that of **diMICAAc** being the lowest. The low Φ_F_ may be advantageously used in cell staining applications where the suppression of background fluorescence is essential. The extended solvatochromic range of the **ICAAc** derivatives can be explained by their increased dipole moment difference between the ground (μ_g_) and excited state (μ_e_) due to the the electron withdrawing isocyano group, which makes the dyes more polar. The dipole moment difference was determined experimentally from the slope of the Lippert-Mataga plot (Fig. S14a) and by DFT calculations. As it is evident from the data of Table 1, the μ_e_-μ_g_ values of the **ICAAc** derivatives are at least 3 times higher than that of AO and are increasing with the methylation of the amino group.

### The behavior of ICAAc derivatives in water

**Figure 3:**
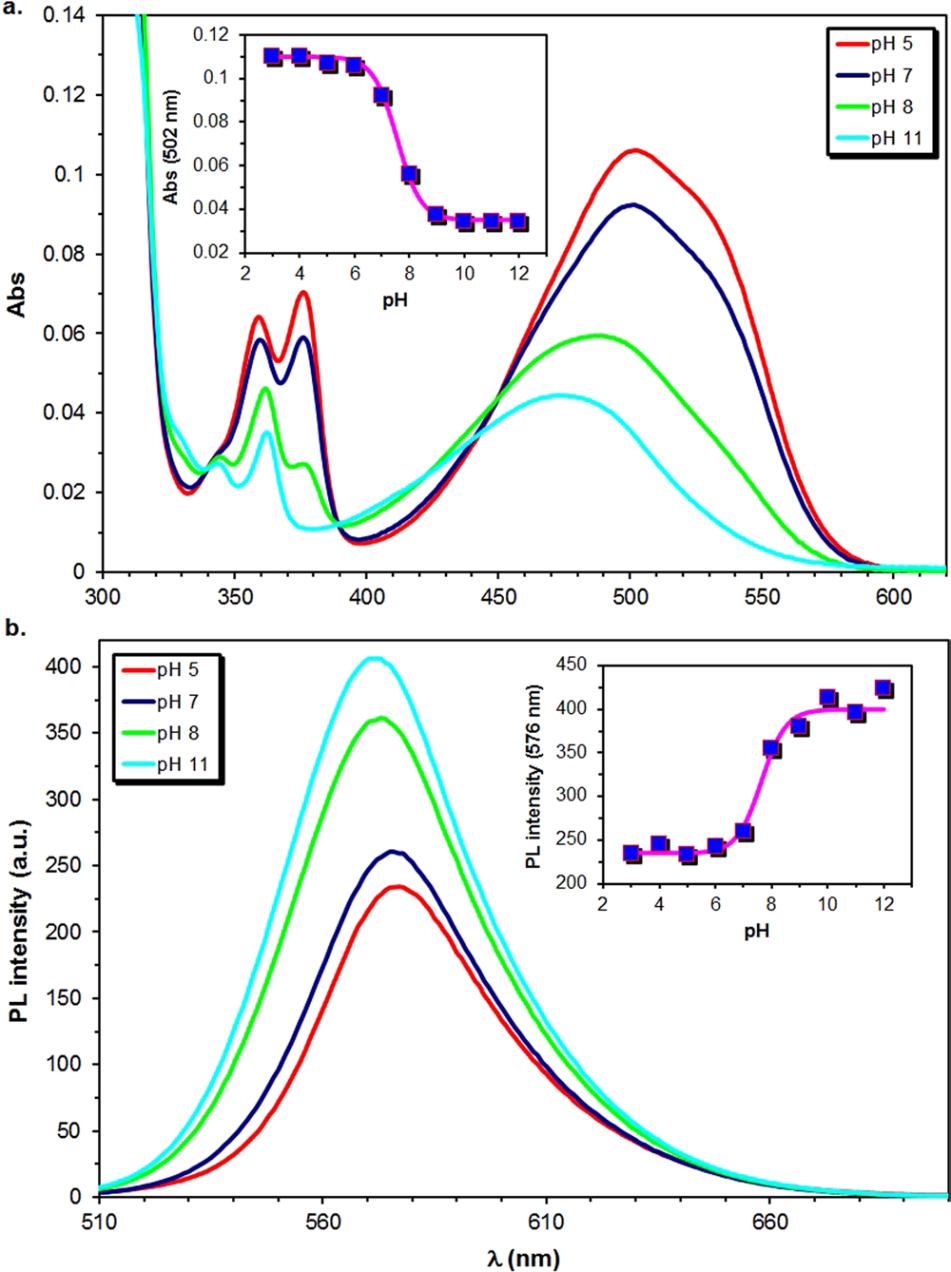
Demonstration of the changes in the UV-vis absorption (**a**) and emission (**b)** spectrum of **diMICAAc** in BRB at different pH. The insets show Eq. 1 fitted on the experimental absorbance or PL intensity data. (T = 20 °C. ([dye] = 4.58 × 10^−5^ M), V = 3.00 cm^3^).

It is known that acridine based dyes such as AO can be present in different forms in aqueous media^22^. To characterize the acid-base properties of our **ICAAc** derivatives UV-vis and steady-state fluorescence measurements were carried out in the pH range of 2–12 in the same Britton–Robinson “universal” buffer (BRB). The results are presented for **diMICAAc** in Fig. 3 and in Figs S16, 17 for **ICAAc** and **monoMICAAc** and are summarized in Table 2.

**Table 2:** Absorption (λ_abs,max_) and emission (λ_em,max_) maxima of the **ICAAc** derivatives at pH = 3 and pH = 11, respectively, in aqueous buffer.

Compound	pH = 3		pH = 11		I_3_/I_11_	pK_a_	pK_a_
	λ_abs,max_	λ_em,max_	λ_abs,max_	λ_em,max_			
	(nm)		(nm)			(UV-vis)	(PL)
***ICAAc***	470	554	428	553	2.25	7.05 ± 0.02	6.96 ± 0.07
***monoMICAAc***	475	553	446	553	1.61	7.35 ± 0.07	7.22 ± 0.15
***diMICAAc***	502	577	470	572	0.58	7.58 ± 0.03	7.71 ± 0.13

I_3_/I_11_ is the fluorescence intensity ratio measured at pH = 3 and pH = 11. pK_a_ is the acid dissociation constant at a logarithmic scale.

The absorption spectrum of all three compounds contains a broad CT band with a maximum of λ_abs,max_ = 470 nm for **ICA**Ac, 475 nm for **monoMICAAc** and 502 nm for **diMICAAc** at pH = 3. This maximum suffers a hypsochromic shift upon increasing the pH of the medium to λ_abs,max_ = 428 nm for **ICAAc**, 446 nm for **monoMICAAc** and 470 nm for **diMICAAc** at pH = 11.

**Figure 4:**
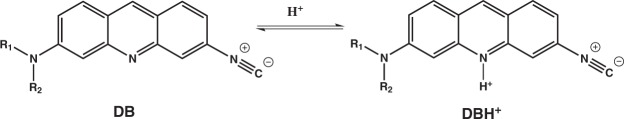
The protonation equilibrium between the free base form DB and the protonated form DBH^+^ of the dyes.

**Figure 5:**
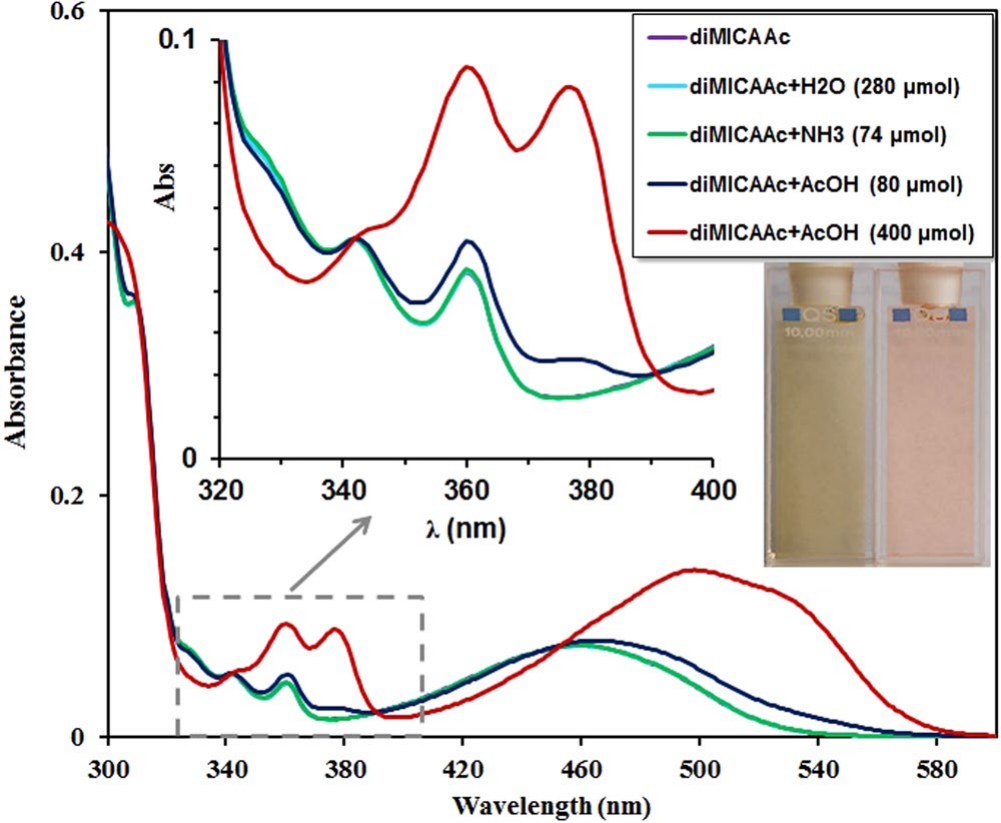
Demonstration of the changes in the UV-vis absorption spectrum of **diMICAAc** dissolved in MeCN upon the addition of water, base (NH_3_) and acid (CH_3_COOH). The inset picture demonstrates the color change of **diMICAAc** upon the addition of acid. Yellow (left, base form), red (right, protonated form).

The base (DB) and protonated (DBH^+^) forms (Fig. 4) can be distinguished by the shape of the minor acridine-like absorption bands located between λ_abs_ = 320 nm and 400 nm. It should be noted that these bands are completely absent from the absorption spectrum of AO, making our dyes more suitable for pH sensing. The UV-vis spectrum of the free base in the same region exhibits a more structured absorption pattern consisting of three moderate bands of different intensity, while the spectrum of the protonated species contains only two broader and higher intensity peaks. This low intensity three-band structure is characteristic for all solvents investigated except water. Therefore, to further support our assignation, UV-vis spectra of **diMICAAc** were recorded in acetonitrile in the presence of water, ammonia and acetic acid (Fig. 5).

According to Fig. 5 the absorption spectra do not change either upon the addition of small amounts of water or base (NH_3_). After the addition of acetic acid in ~7 molar excess, a new band starts to appear at ~375 nm and a broad double band structure only appears after the addition of a large excess (~35x molar) of acetic acid. After recording the absorption spectra in the concentration range of 10^−6^–10^−4^ M in water and in buffers too, no deviation from the Lambert-Beer law was observed, which excludes the presence of dimers.

**Figure 6:**
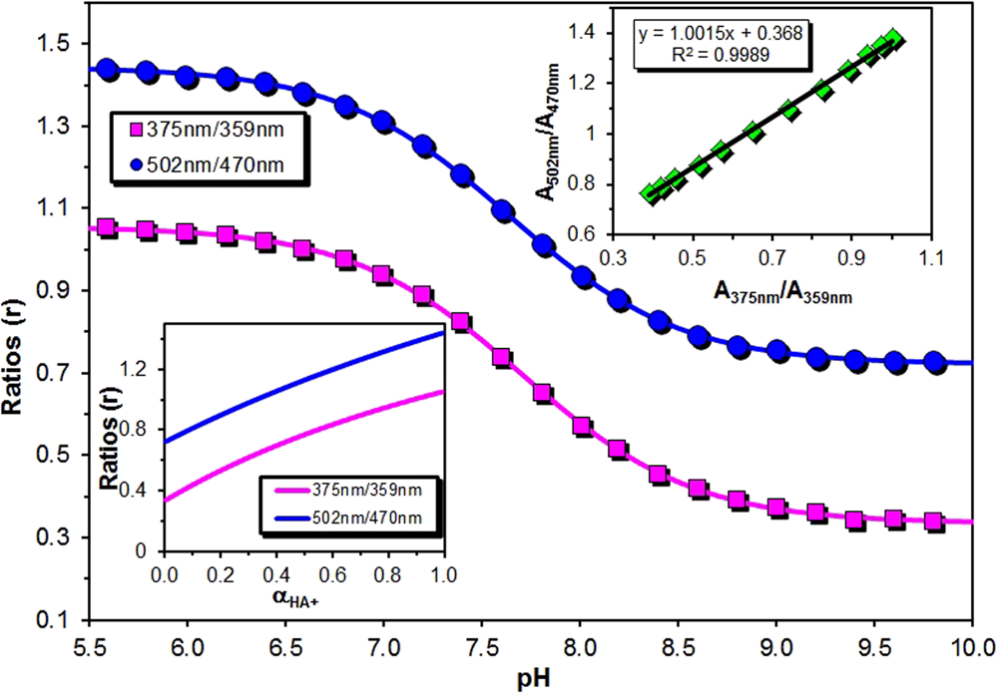
UV-vis titration curves of **diMICAAc** in BRB buffer at different pHs. The lines were fitted using Eq. 2. The bottom left inset shows the absorbance ratios as a function of the molar fraction of the protonated form α_HA+_. The top right inset shows the correlation between the absorbance ratios calculated at the ICT and acridine like absorption wavelengths. (T = 20 °C, [dye] = 1.14 × 10^−5^ M, V = 3.00 cm^3^).

Assuming a simple equilibrium between DB and DBH^+^ (Fig. 4) and denoting the pH-dependent property (absorbance or PL intensity) by *Y(λ,pH)*, the acid dissociation constant (K_a_) can be determined by fitting Eq. 1. to the Abs(502)-pH experimental plots as presented in the inset of Fig. 6.

1 $$Y(\lambda ,pH)=\frac{{Y}_{B}(\lambda )}{1+{10}^{p{K}_{a}-pH}}+\frac{{Y}_{BH+}(\lambda )\cdot {10}^{p{K}_{a}-pH}}{1+{10}^{p{K}_{a}-pH}}$$

The obtained pK_a_ values show only a slight variation between pK_a_ = 7.05 and 7.58 (UV-vis) and pK_a_ = 6.96 and 7.71 (Fluorescence). These pK_a_ values make the **ICAAc** derivatives three orders of magnitude weaker bases than AO (pK_a_ = 10.5)^22^. The fluorescence intensity also shows strong pH dependence, it increases in the more acidic medium in the case of **ICAAc**, and **monoMICAAc**. The PL intensity ratio I_3_/I_11_ calculated from the intensities measured at pH = 3 and pH = 11 is 2.25 and 1.69 for **ICAAc**, and **monoMICAAc**, respectively. The situation is completely different for **diMICAAc**, where PL intensity increases at higher pH and I_3_/I_11_ is only 0.58.

### Investigation of the applicability of diMICAAc for pH determination

Favorably, the pK_a_ value of **diMICAAc** is 7.5, which falls in the physiological pH range making it a possible candidate for a fluorescent biosensor. For the precise determination of the pH, calibration curves were constructed based on Eq. 2.

2 $$r=\frac{{b}_{1}+{10}^{-pH}}{{b}_{2}+{b}_{3}{10}^{-pH}}$$

where r is the ratio of the absorbances (PL intensities) measured at different wavelengths, b_1_, b_2,_ and b_3_ are parameters used for fitting. The UV-vis titration curves are presented in Fig. 6 and the fluorescence titration curves in Fig. S18.

As it is evident from Fig. 6 both curves have a very similar run. The A_502_/A_470_ ratio varies between 1.43–0.73, while the A_375_/A_359_ between 1.05–0.34 in the pH range of 5.5–10.0. The majority of the change happens between pH = 6.5–8.5, making it the most useful region for pH determination. In addition, it should be noted that there is a perfect correlation between the two absorbance ratios as is presented in Fig. 6 inset. Unlike UV-vis, the fluorescence (excitation) titration curve is shifted approximately 1 pH unit to the left, that is the drop in the curve is located between pH = 5.5–8.0, extending the useful section more in the acidic region. To test the practical applicability of our dye and the reproducibility of the method, that is its dependence on the dissolved chemical species and ionic strength, buffer media of different pH and concentration were prepared and their absorption and fluorescence spectra were recorded after the addition of **diMICAAc**. The results are summarized in Table 3.

**Table 3:** Reproducibility of the spectroscopic pH sensing method using different buffers.

A_375_/A_359_	I_376_/I_362_	pH_Set_	pH_Abs_	pH_PL_
1.031 ± 0.003 *1.043* ± *0.002*	0.986 ± 0.006 *1.005* ± *0.008*	6.01	6.24 ± 0.06 *5.98* ± *0.002*	6.06 ± 0.03 *5.97* ± *0.04*
1.000 ± 0.002 *1.020* ± *0.011*	0.814 ± 0.004 *0.857* ± *0.008*	6.49	6.63 ± 0.01 *6.42* ± *0.15*	6.55 ± 0.01 *6.45* ± *0.02*
0.914 ± 0.003 *0.934* ± *0.004*	0.562 ± 0.002 *0.623* ± *0.002*	7.00	7.10 ± 0.01 *7.02* ± *0.02*	7.06 ± 0.00 *6.94* ± *0.00*
0.772 ± 0.011 *0.751* ± *0.006*	0.367 ± 0.019 *0.365* ± *0.001*	7.51	7.52 ± 0.03 *7.57* ± *0.02*	7.55 ± 0.07 *7.56* ± *0.00*
0.589 ± 0.006 *0.565* ± *0.002*	0.272 ± 0.001 *0.267* ± *0.002*	8.00	7.97 ± 0.02 *8.03* ± *0.05*	8.03 ± 0.01 *8.08* ± *0.02*
0.445 ± 0.009 *0.440* ± *0.007*	0.234 ± 0.001 *0.231* ± *0.001*	8.50	8.45 ± 0.04 *8.47* ± *0.03*	8.54 ± 0.03 *8.60* ± *0.02*
0.395 ± 0.002 *0.377* ± *0.006*	0.219 ± 0.002 *0.219* ± *0.001*	9.00	8.75 ± 0.02 *8.91* ± *0.06*	9.17 ± 0.23 *9.19* ± *0.11*

NaH_2_PO_4_/Na_2_HPO_4_ (normal text) NaH_2_BO_3_/Na_2_HBO_3_ (italic text). **pH**_**Set**_, **pH**_**Abs**,_ and **pH**_**PL**_ are the pH(s) determined using a pH meter, from the UV-vis and the fluorescence spectrum, respectively. c_buffer_ = 0.05 M in both cases.

The spectroscopically measured pH values for both the phosphate and borate buffers agree well with the previously set values in the 6.00–9.00 pH region. No selectivity for different chemical species could be detected. The largest errors 2–4% can be observed on the edges of the range, whereas between pH = 7.00–8.50 the errors are well below 1% for both the UV-vis and the fluorescence methods, which is important for biological applications. The method was also tested on a 0.10 M NaHCO_3_ solution with a pH of 8.50 and the measured values were pH = 8.53 ± 0.07 (UV-vis) and pH = 8.59 ± 0.01 (PL). The stability of the method was also tested in phosphate buffer at pH = 6 and 8 respectively for an hour. No significant change (the relative errors were 0.6% at pH = 6 and 0.4% at pH = 8) could be observed during this period as can be seen in Figs S19, 20. To investigate the salt effect, samples in BRB buffer at pH = 6 and 8, respectively were titrated with saturated KCl solution. As Fig. S21 shows the ionic strength has no significant effect on the method, the errors are below 1–2% even at 0.5 M KCl concentrations. Hence, we can conclude that the method is stable and can be used as soon as 5 minutes after mixing the dye with the medium.

### Critical micelle concentration (CMC) studies

**Figure 7:**
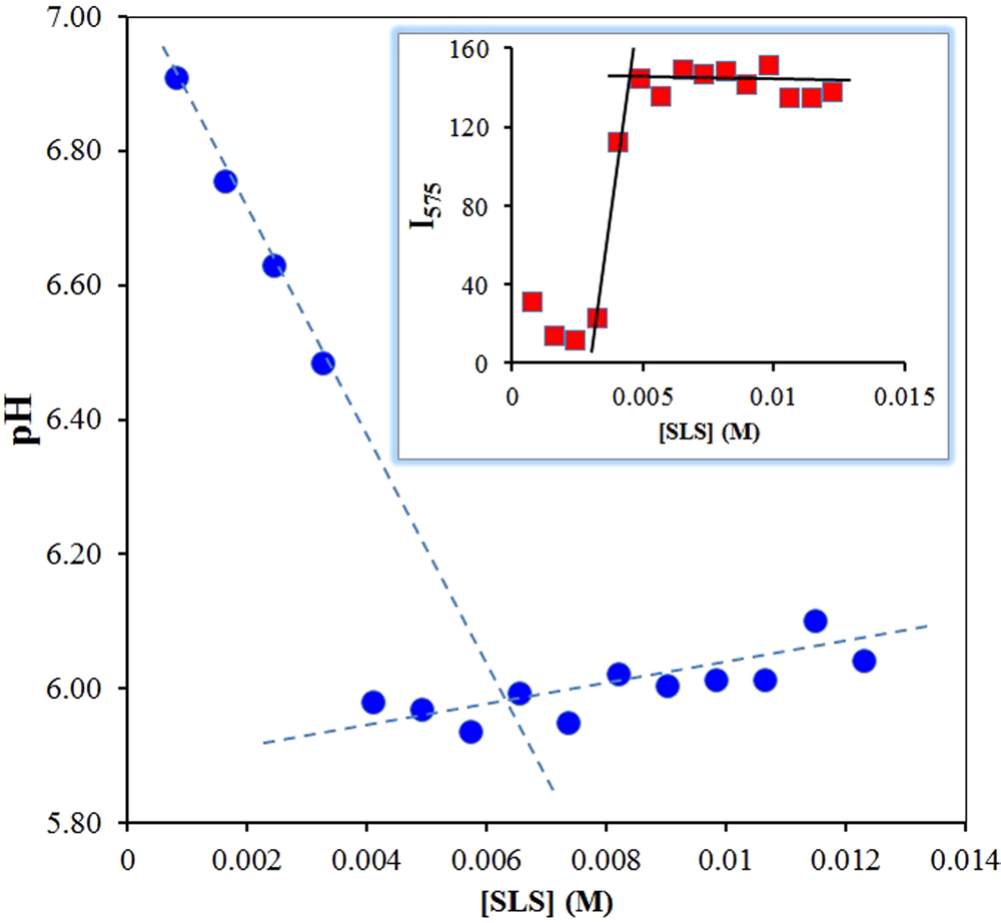
Calculated pH of the microenvironment of **diMICAAc** *versus* SLS concentration in BRB (pH = 7). The inset shows the fluorescence intensities measured at λ = 575 nm versus SLS concentration (T = 20 °C, [dye] = 4.58 × 10^−6^ M, V = 3.00 cm^3^).

It has been shown previously that the pH in the Stern layer of micelles formed from anionic surfactants such as sodium lauryl sulfate (SLS) can significantly differ from that of the bulk^28^. It is known that AO interacts with SLS micelles through both hydrophobic and electrostatic interactions. Since our dyes are highly dipolar and the intracyclic imino group gets easily charged through protonation, it is more likely that the dyes will reside in the Stern layer instead of penetrating into the nonpolar core. In order to investigate the behavior of our dyes in the micellar medium, we have studied **diMICAAc** in SLS solutions prepared in 50 mM BRB buffer at pH = 7 (to keep the bulk pH constant). The pH values were calculated from the excitation spectra using the ratio of I_376_/I_362_ and the fluorescence intensities were read at 575 nm (peak maximum). The results are summarized in Fig. 7. As it is evident from Fig. 7 the pH of the microenvironment of **diMICAAc** starts to decrease in the first four steps by increasing the SLS concentration. This linear decrease is followed by a sharp drop to pH = 6 and then fluctuates around this value. Since Φ_F_ = 0.4% for **diMICAAc**, the interaction with micelles results in a sharp increase in fluorescence intensity at 575 nm. The change in pH and PL intensity *vs* concentration curves correlate perfectly, the sharp drop (jump) happens at approximately 4 mM which can be considered the critical micelle concentration (CMC) in this medium. However, this value is a bit lower than the one reported in pure water, it is known that the use of a buffer solution, therefore the elevated ionic strength results in a lower CMC^36^.

The experiments were repeated using deionized water and **diMICAAc** and the results are presented in Fig. S22. Similarly to the buffered solution, below CMC the PL intensity decreases until a minimum is reached followed by a sudden increase after which the PL intensity runs into saturation above CMC. It is evident from Fig. S22b. that the run of the intensity vs SLS concentration curves are identical and no matter which wavelength we use the same CMC value of approximately 7 mM is obtained, which is in good agreement with the values found in the literature for SLS. When looking at Fig. S22a. we can see the variation of the excitation spectrum as a function of SLS concentration. It should be emphasized that independently from the wavelengths used, the CMC of SLS can be very well guessed from the excitation spectra too. At 5.74 and 6.54 mM SLS concentrations (indicated by thick lines) we can see the shape of the spectra alter, that is a shoulder at 530 nm appears on the ICT band and it almost becomes a double band. This change can be assigned to the start of the increasing part of the intensity curve in the premicellar region of SLS concentration. Above CMC the 530 nm shoulder remains, but the 503 nm band becomes dominant. It should be noted that λ_em,max_ does not change above or below CMC with respect to the one recorded in pure water. Therefore the dye is not likely to penetrate to the nonpolar core of the micelles and it most likely resides in the Stern layer of the micelles where the interaction is of electrostatic character. For comparison, the CMC was also determined using the more sophisticated method of Pineiro *et al*.^37^, where the CMC is defined as the total surfactant concentration [S]_0_ at which the change in the gradient of the monomer concentration [S_1_] with respect to [S]_0_ is maximum, that is where the third derivative of [S_1_] is zero. Consequently, we describe the second derivative of [S_1_] with respect to [S]_0_ by a Gaussian function centered at the CMC as is presented in Fig. S23. The CMC obtained by this method is 7.5 mM which is very close to the literature value of 8.2 mM. In summary, **diMICAAc** is well applicable for both CMC determination and microenvironment pH mapping.

### Complexation of diMICAAc with Ag(I) ions

**Figure 8:**
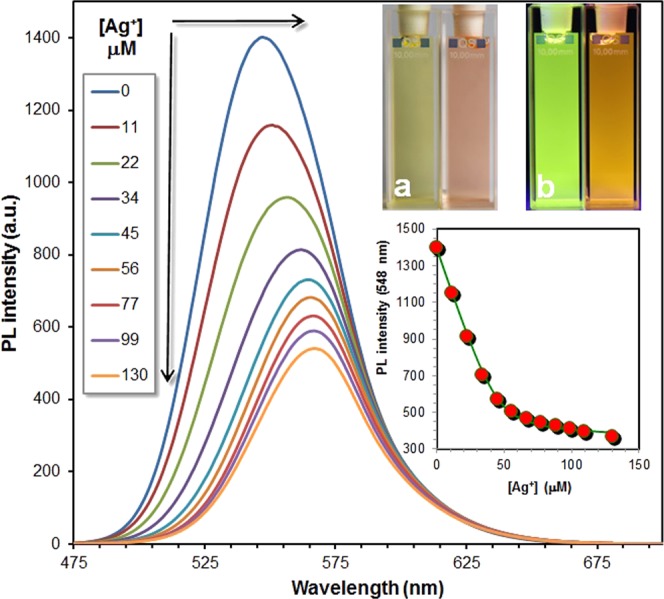
The change of fluorescence as a function of metal (Ag^+^) concentration in the dioxane solution of **diMICAAc** (4.58  ×  10^−5^ M). Picture (**a**) **diMICAAc** in dioxane (left) and in the presence of 130 μM Ag^+^ (right) under visible light. Picture (**b**) the same compositions as in (**a**) illuminated by λ_ex_ = 365 nm UV-light.

We have shown earlier^34^ that aromatic amino-isocyano compounds tend to form complexes in the presence of Ag(I) ions and the process is accompanied by significant spectral changes. Steady-state fluorescence titration of **diMICAAc** in dioxane revealed a sharp decrease of PL intensity (to one-third of the original) with increasing silver(I) concentration as is presented in Fig. 8. In addition, a 20 nm bathochromic shift of the emission maximum occurs from λ_em,max_ = 548 nm to λ_em,max_ = 568 nm, which manifests in a color change from greenish-yellow to red in visible light and from neon green to orange when excited with λ_ex_ = 365 nm UV light (Fig. 8 insets a and b respectively). The shape of the inset graph in Fig. 8 indicates the formation of a 1:1 complex, which was also detected under ESI-MS conditions. The first isotopic mass of the complex with a formula of [Ag(C_16_H_13_N_3_)]^+^ is 354.015 Da as can be seen in Fig. S24 and no additional mass corresponding to other Ag:**diMICAAc** composition could be observed. The equilibrium constant of complexation was determined using non-linear regression analysis described in ref.^34^ and turned out to be K = (4.5 ± 0.9) × 10^5^ M ^−1^. However, this equilibrium constant is lower than those obtained for ICAN derivatives (*K* = 10^6^ M^−1^ and 10^7^ M^−1^) it still indicates high binding affinity of silver(I) towards **diMICAAc**. The formation of a 1:1 complex is further backed by quantum chemical (DFT) calculations since the calculated PL spectrum agrees very well with the measured one as can be seen in Fig. S25. DFT calculations also revealed that Ag^+^ is attached to the isocyano moiety instead of the imino nitrogen in the ring (Fig. S26).

### Computational results

**Figure 9:**
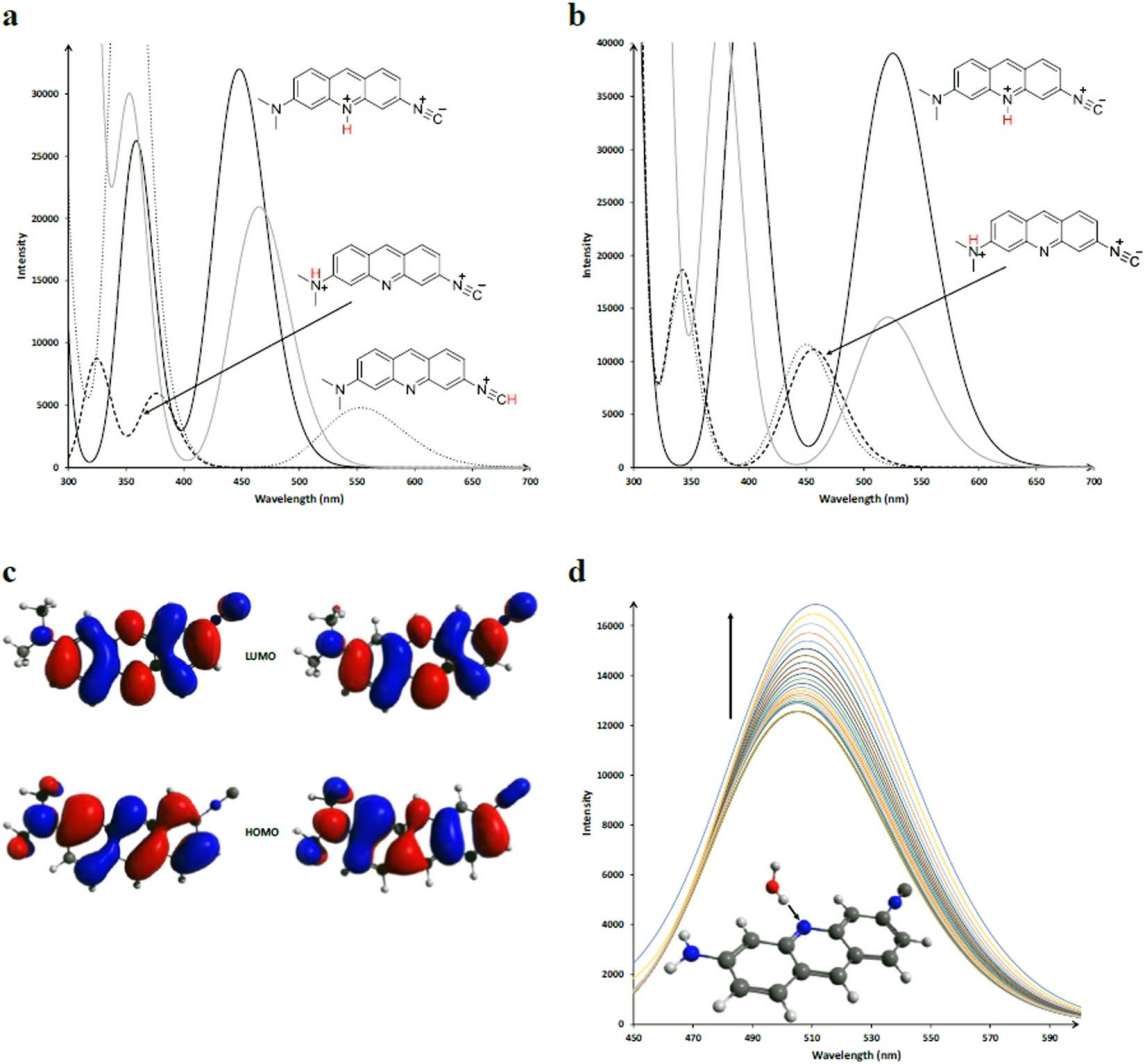
(**a**) The calculated UV-Vis absorption spectra of the three different protonation modes of **diMICAAc** in water. The relative Gibbs free energies in the ground state are the following: 0, +17.4 and +26.7 kcal mol^−1^ for the acridine N and amino N, and isocyano C protonated states, respectively. (**b**) The calculated emission spectra of the amino and acridine N protonation modes of **diMICAAc** in water. The aminomethyl groups are perpendicular to the acridine ring. The relative electronic energies in the excited state are the following: 0, +17.3 for the acridine N, amino N protonated states, respectively. (**c**) The frontier orbitals of **diMICAAc** (left) and its acridine N protonated (right) form. (**d**) Change in the emission spectrum when the acridine nitrogen gets protonated along a relaxed potential energy scan of the H_water_ and N_acridine_ distance. The protonated state is not reached in the end.

The calculated absorption and emission spectra of the three amino-isocyanoacridine molecules are shown in Figs S27–32. In nonpolar solvents, the calculated and experimental spectra show very good agreement. Considering polar solvents, however, it seems that additional effects, other than simple dielectric interactions play an important role. First, we investigated whether the observed fluorescence quenching is due to the decoupling of either the amino or the isocyano group from the acridine ring by simply replacing the two functional groups with a hydrogen atom. As Fig. S33 shows, both spectra are blue-shifted compared to the unmodified **ICAAc** but no dark states or lower oscillator strengths are calculated. The TICT (Twisted Intramolecular Charge Transfer) mechanism is unlikely because the planarization of the amino group in the excited state is not hindered by nearby hydrogens according to geometry optimization in the excited state. Another commonly reported quenching mechanism, the dimerization is also not expected to play a role because dimer formation of **ICAAc** is endergonic by 3.3–5.0 kcal mol^−1^. Since these molecules can be used as pH sensors, we also explored the possible sites of protonation, as well as the optical properties of these protonated species using DFT. We considered three protonation modes, as shown in Fig. 9a,b. It can be seen, that in both the ground and S1 states, the protonation of the acridine nitrogen is by far the most favored case. Protonation of the amino group is also interesting because it gives rise to the observed double peak structure below 400 nm in the absorption spectrum but the low energy band disappears. The change in the experimental fluorescence spectrum of **ICAAc** upon protonation is reproduced by the calculations well, however, the emission behavior of **diMICAAc** remains an open question. As Fig. 9c shows, the frontier orbitals barely change upon protonation which is reflected by the very small change in the calculated emission spectra. We also considered another approach where an excited state proton transfer was modeled using an explicit water molecule. The spectral changes following the gradual decrease of the N-H distance are shown in Fig. 9d. This shows the experimentally observed behavior of a minimal red shift together with a nearly twofold increase in intensity. Correlation between the protonation coordinate and change in emission wavelength for the protonation and deprotonation of the amino group is shown in Figs S34, 35. The calculated wavelength shift is in the opposite direction which further strengthens the view that protonation takes place on the acridine nitrogen.

### Overview of the staining capacity of the new dyes for fluorescence microscopy and cytometry in live human cells

The biological applicability of the dyes as supravital cell stains (Laser-Scanning Cytometry and microscopy) and the investigation of their toxicity (MTT cell viability assay) are presented in the Supporting Information as Chapter VII. Biological studies.

## Conclusions

Novel, multifunctional, amino-isocyanoacridines (**ICAAc**, **monoMICAAc** and **diMICAAc**) were prepared by the reaction of 3,6-diaminoacridine with dichlorocarbene. The resulting dyes showed tunable solvatochromic behavior (Δλ_em_ = 53–63 nm) owing to the dipolar nature (μ_e_-μ_g_ varies between 5.6–8.9 Debye) of the molecules. The introduction of the electron withdrawing isonitrile group, resulted in a reduced basicity (pK_a_ = 7.05–7.58), which in combination with their unique pH-sensitive vibronic absorption bands make them promising pH-probes in the physiological pH range. It was demonstrated using UV-vis and fluorescence spectroscopy that the pH of different buffer solutions could be recovered with great precision (2–3% error) between pH = 6–8 and the method is stable for at least an hour. The dyes were shown to be able to sense pH change in different microenvironments such as the lowered pH in the Stern layer as it was demonstrated on SLS micelles. Furthermore, the isocyano group is an excellent ligand for complexation with Ag(I), this way the optical behavior of our dyes can be fine-tuned by ions too. 24 hour MTT assays on HeLa cells revealed LD_50_ values of 7.27, 5.78, and 7.50 μM for **ICAAc**, **monoMICAAc** and **diMICAAc** dyes, respectively. The applicability of **ICAAc**, **monoMICAAc**, and **diMICAAc** dyes for live cell imaging was demonstrated on HeLa cells at 0.3 μg/ml dye concentration. **diMICAAc** stained cells demonstrated the best-preserved morphology. **DiMICAAc** probably binds cell membranes, some unknown intracellular vesicular structures, probably the DNA slightly, and possible binds to some extent to the RNA. All the three dyes are well-applicable with conventional epifluorescence imaging at the UV excitation. Furthermore, at the blue excitation, **diMICAAc** is optimally suited as a whole-cell probe for both the conventional microscopic and the laser-illumination studies, like flow- and imaging cytometric, or confocal laser-scanning microscopic examinations.

## Methods

### Materials

Acetone, dichloromethane (DCM), hexane, 2-propanol (iPrOH), toluene, ethyl-acetate (EtOAc) (reagent grade, Molar Chemicals, Hungary) were purified by distillation. Acetonitrile (MeCN), tetrahydrofuran (THF), methanol (MeOH), dimethyl formamide (DMF), dimethyl sulfoxide (DMSO), pyridine (HPLC grade, VWR, Germany), cyclohexane, 1,4-dioxane (reagent grade, Reanal, Hungary), chloroform, 3,6-diaminoacridine hydrochloride (Sigma-Aldrich, Germany), sodium lauryl sulfate (SLS) (Biosolve, France) were used without further purification.

### Synthesis

The preparation and characterization of the **ICAAc** derivatives are detailed in the SI.

### Methods

#### UV-vis

The UV-vis spectra were recorded on an Agilent Cary 60 spectrophotometer (Agilent, Santa Clara, CA, USA) in a quartz cuvette of 1.00 cm optical length. 3.00 cm^3^ solution was prepared from the sample.

#### Fluorescence measurements

Steady-state fluorescence measurements were carried out using a Jasco FP-8200 fluorescence spectrophotometer equipped with a Xe lamp light source. The excitation and emission spectra were recorded at 20 °C, using 2.5 nm excitation, 5.0 nm emission bandwith, and 200 nm/min scanning speed. Fluorescence quantum yields (Φ) were calculated by using quinine-sulfate as the reference, using the following equation:

3 $${\rm{\Phi }}={\rm{\Phi }}r\,\ast \,\frac{I}{{I}_{ref}}\,\ast \,\frac{{A}_{ref}}{A}\,\ast \,\frac{{n}^{2}}{{n}_{ref}^{2}}$$

where Φ_r_ is the quantum yield of the reference compound (quinine-sulfate in 0.1 mol/L sulfuric acid, absolute quantum efficiency (Φ_r_ = 55%)), *n* is the refractive index of the solvent, *I* is the integrated fluorescence intensity and *A* is the absorbance at the excitation wavelength. The absorbances at the wavelength of excitation were kept below *A* = 0.1 in order to avoid inner filter effects. For UV-vis and fluorescence measurements the investigated compounds were dissolved in acetonitrile at a concentration of 1.2 mM and was diluted to 2.4 × 10^−5^ M and 4.8 × 10^−6^ M in the solvents in interest.

#### Measurements on the pH-dependence of the optical properties

Britton-Robinson buffer (BRB) was prepared by mixing 100–100 cm^3^ 0.04 M H_3_BO_3_, 0.04 M H_3_PO_4_ and 0.04 M acetic acid solution, then titrated to the desired pH with 2.0 M NaOH solution. The cuvette was charged with 3.00 cm^3^ buffer solution, and 30 μl stock solution of the dye in acetonitrile was added. NaH_2_PO_4_/Na_2_HPO_4_ and NaH_2_BO_3_/Na_2_HBO_3_ buffers were prepared by the titration of 0.05 M Na_2_HPO_4_ or Na_2_B_4_O_7_ solution with 1.0 M NaOH or HCl to set the desired pH.

#### CMC studies

A stock solution of sodium lauryl sulfate (SLS) was prepared in water or 0.004 M pH = 7.01 BRB buffer at a concentration of 0.082 M, and was diluted to the given concentration. The cuvette was charged with 3.00 cm^3^ SLS solution, and 12 μl stock solution of the dye in acetonitrile was added. Optical properties were measured after 1 hour equilibration.

#### Complexation of diMICAAc with Ag(I) ions

The cuvette was charged with 3.00 cm^3^ dioxane and 120 μl stock solution of the dye in acetonitrile, then silver trifluoroacetate stock solution (3.4 mM in dioxane) was added in 10 μl portions.

*Laser-Scanning Cytometry and microscopy and MTT cell viability assay* are presented in the Supporting Information.

## Supplementary information

Amino-isocyanoacridines: Novel, Tunable Solvatochromic Fluorophores as Physiological pH Probes

## Data Availability

All data generated or analysed during this study are included in this published article (and its Supplementary Information Files) or are available from the corresponding author on reasonable request.
