# Supplementary material for: Amino-isocyanoacridines: Novel, Tunable Solvatochromic Fluorophores as Physiological pH Probes
Source: Sci Rep. 2019 Jun 3;9:8250. doi: 10.1038/s41598-019-44760-9 (PMC6547668; doi:10.1038/s41598-019-44760-9)
Supplement: Supplementary file 1 — Amino-isocyanoacridines: Novel, Tunable Solvatochromic Fluorophores as Physiological pH Probes [file 41598_2019_44760_MOESM1_ESM.docx]

**Amino-isocyanoacridines: Novel, Tunable Solvatochromic Fluorophores as Physiological pH Probes**

Miklós Nagy^1^, Dávid Rácz^1^, Zsolt László Nagy^1^, Péter Pál Fehér^2^, Sándor Lajos Kovács^1^, Csaba Bankó^3^ , Zsolt Bacsó^3^, Alexandra Kiss^4^, Miklós Zsuga^1^, Sándor Kéki^1^*

^1^*Department of Applied Chemistry, ^4^Department of Biotechnology and Microbiology, Faculty of Science, University of Debrecen, 4010, Hungary*

*^2^Research Centre for Natural Sciences, Hungarian Academy of Sciences, H-1117 Budapest, Magyar tudósok körútja 2, Hungary*

*^3^University of Debrecen, Medical and Health Science Center, Faculty of Medicine, Department of Biophysics and Cell Biology, 4010, Hungary*

**Supporting information**

**Table of Contents**

[Synthesis of ICAAc derivatives 5](#_Toc515297540)

[3-amino-6-isocyanoacridine (ICAAc) 5](#_Toc515297541)

[3-N-methylamino-6-isocyanoacridine (monoMICAAc) and 3-N,N-dimethylamino-6-isocyanoacridine (diMICAAc) 5](#_Toc515297542)

[Chapter I. 3-amino-6-isocyanoacridine (ICAAc) 7](#_Toc515297543)

[**Figure S1.** ^1^H-NMR and ^13^C-NMR spectra of ICAAc 7](#_Toc515297544)

[**Figure S2.** The measured and calculated ESI-MS spectra of ICAAc 8](#_Toc515297545)

[Experimental conditions of Electrospray Quadrupole Time-of-Flight MS/MS (ESI-Q-TOF) 8](#_Toc515297546)

[**Figure S3.** UV-Vis absorbance spectrum of of ICAAc recorded in different solvents 9](#_Toc515297547)

[**Figure S4.** Fluorescence emission and excitation spectra of ICAAc recorded in different solvents 10](#_Toc515297548)

[Chapter II. 3-N-methylamino-6-isocyanoacridine (monoMICAAc) 12](#_Toc515297549)

[**Figure S5.** ^1^H-NMR and ^13^C-NMR spectra of monoMICAAc 12](#_Toc515297550)

[**Figure S6.** The measured and calculated ESI-MS spectra of monoMICAAc 13](#_Toc515297551)

[**Figure S7.** UV-Vis absorbance spectrum of monoMICAAc recorded in different solvents 14](#_Toc515297552)

[**Figure S8.** Fluorescence emission and excitation spectra of monoMICAAc recorded in different solvents 15](#_Toc515297553)

[Chapter III. 3-N,N-dimethylamino-6-isocyanoacridine (diMICAAc) 17](#_Toc515297554)

[**Figure S9.** ^1^H-NMR and ^13^C-NMR spectra of diMICAAc 17](#_Toc515297555)

[**Figure S10.** The measured and calculated ESI-MS spectra of diMICAAc 18](#_Toc515297556)

[**Figure S11.** The measured and calculated ESI-MS spectra of [Ag(diMICAAc)]^+^ 18](#_Toc515297557)

[**Figure S12.** UV-Vis absorbance spectrum of diMICAAc recorded in different solvents 19](#_Toc515297558)

[**Figure S13.** Fluorescence emission and excitation spectra of diMICAAc recorded in different solvents 20](#_Toc515297559)

[Chapter IV. Environment-sensitivity of the fluorophores 22](#_Toc515297560)

[Lippert-Mataga equation 22](#_Toc515297561)

[**Figure S14.** Demonstration of the variation of the photophysical properties of monoMICAAc as a function of solvent polarity: (**a**) Stokes shift *vs* orientation polarizability (Δf) (Lippert-Mataga plot), (**b**) emission maximum *vs* solvent polarity parameter E_T_(30) (**c**) quantum yield *vs* E_T_(30). 22](#_Toc515297562)

[**Figure S15.** Demonstration of the variation of the photophysical properties of ICAAc and diMICAAc as a function of solvent polarity 23](#_Toc515297563)

[**Figure S16.** Demonstration of the changes in the UV-vis absorption and emission spectrum of ICAAc in Britton–Robinson „universal” buffer at different pH 24](#_Toc515297564)

[**Figure S17.** Demonstration of the changes in the UV-vis absorption and emission spectrum of monoMICAAc in Britton–Robinson „universal” buffer at different pH. 25](#_Toc515297565)

[**Figure S18.** Fluorescence (excitation) titration curves of diMICAAc in Britton–Robinson buffer at different pHs 26](#_Toc515297566)

[**Figure S19.** Stability test of the fluorescence of diMICAAc in phosphate buffer at pH=6 2](#_Toc515297568)7

[**Figure S20.** Stability test of the fluorescence of diMICAAc in phosphate buffers at pH=6 and 8. 2](#_Toc515297569)8

**Figure S21**. The effect of salt on the fluorescence of diMICAAc in BRB buffers at pH=6 and 8. 28

[**Figure S22** Fluorescent spectral changes of **diMICAAc** versus SLS concentration in water: (**a**) excitation spectra (**b**) PL Intensity maxima obtained from the emission and from the excitation spectra *vs* SLS concentration 29](#_Toc515297570)

[**Figure S23.** Variation of the intensity of the excitation peak at 375 nm as a function of SLS concentration 30](#_Toc515297571)

[Chapter V. Metal complexation studies 31](#_Toc515297572)

[**Figure S24.** The measured and calculated ESI-MS spectra of Ag[(C_16_H_13_N_3_)]^+^ or Ag:diMICAAc 1:1 complex 31](#_Toc515297573)

[**Figure S25.** The calculated emission spectra of diMICAAc and Ag:diMICAAc 1:1 complex in dioxane. 32](#_Toc515297574)

[**Figure S26.** The calculated UV-vis absorption spectra of the two Ag:diMICAAc complexes together with the diMICAAc 33](#_Toc515297575)

**Figure S27.** The calculated UV-Vis absorption spectra of the three different protonation modes of diMICAAc in water 36

[Chapter VI. Computational results 34](#_Toc515297576)

[**Figure S28.** The calculated UV-vis absorption spectra of ICAAc in different solvents 3](#_Toc515297577)5

[**Figure S29.** The calculated emission spectra of ICAAc in different solvents. 3](#_Toc515297578)6

[**Figure S30.** The calculated UV-Vis absorption spectra of monoMICAAc in different solvents 3](#_Toc515297579)7

[**Figure S31.** The calculated emission spectra of monoMICAAc in different solvents](#_Toc515297580) 38

[**Figure S32.** The calculated UV-Vis absorption spectra of diMICAAc in different solvents](#_Toc515297581) 39

[**Figure S33.** Emission spectra of the truncated ICAAc molecule 4](#_Toc515297582)0

[**Figure S34.** Relaxed PES scan of the N-H distance (protonation of the amino group) in the S1 state 4](#_Toc515297584)1

[**Figure S35.** Relaxed PES scan of the O-H distance (deprotonation of the amino group) in the S1 state 4](#_Toc515297585)2

[Computational Details 4](#_Toc515297586)3

[Chapter VII. Biological studies 44](#_Toc515297587)

[Cell line 4](#_Toc515297588)8

[Laser-Scanning Cytometry and microscopy 4](#_Toc515297589)8

[MTT cell viability assay 4](#_Toc515297590)9

[**Figure S36.** Demonstration of the staining capacity of **ICAAc** derivatives for live cell imaging in HeLa cells. 45](#_Toc515297595)

[**Figure S37.** Montage of well images captured by an iCys imaging cytometer to evaluate fluorescence staining and acute toxicity of the three dyes 47](#_Toc515297595)

[**Figure S38.** Results of the dye toxicity test employing the colorimetric cell viability MTT test in a 96-well plate on HeLa cells 50](#_Toc515297591)

[**Figure S39.** Viability curve of the ICAAc 5](#_Toc515297592)1

[**Figure S40.** Viability curve of the monoMICAAc 5](#_Toc515297593)2

[**Figure S41.** Viability curve of the diMICAAc 5](#_Toc515297594)3

# Synthesis of ICAAc derivatives

### 3-amino-6-isocyanoacridine (ICAAc)

In a 250 ml round-bottom flask 3,6-diaminoacridine hydrochloride (1.00 g, 4.07 mmol) and potassium hydroxide (2.28 g, 40.7 mmol) suspended in 50 ml chloroform and 50 ml dichloromethane was stirred for 30 minutes, then extracted with water, the organic phase was dried over anhydrous magnesium sulfate, and the solvent was removed on a rotary evaporator. The crude product was purified on a column filled with normal-phase silica gel, using dichloromethane:methanol:acetone 90:5:5 as eluent. Yield: 0.31 g, 32 % (orange crystals).

^1^H NMR (360 MHz, DMSO) δ= 8.77 (s, 1H), 8.13 – 7.99 (m, 2H), 7.86 (d, *J* = 9.0 Hz, 1H), 7.44 (d, *J* = 8.7 Hz, 1H), 7.16 (d, *J* = 9.0 Hz, 1H), 6.92 (s, 1H), 6.36 (s, 2H) ppm.

^13^C NMR (95 MHz, DMSO) δ = 165.70 (C_NC_), 152.64 (C_3_), 152.37 (C_5a,10a_), 148.16 (C_6_), 135.92 (C_9_), 131.39 (C_8_), 130.11 (C_1_), 125.68 (C_7_), 123.94 (C_9a_), 122.51 (C_5_), 122.04 (C_8a_), 120.36 (C_2_), 102.68 (C_4_) ppm.

ESI-TOF MS (m/z): calculated m/z of [C_14_H_9_N_3_ + H]^+^ adduct ion is 220.087; found; 220.088.

### 3-N-methylamino-6-isocyanoacridine (monoMICAAc) and 3-N,N-dimethylamino-6-isocyanoacridine (diMICAAc)

A 250 ml round-bottomed flask, equipped with a magnetic stirbar was charged with 3-amino-6-isocyanoacridine (1.00 g, 4.80 mmol), potassium hydroxide (2.96 g, 52.8 mmol) and absolute toluene freshly distilled over sodium (50 ml). Methyl iodide (2.99 ml, 48.0 mmol) was added to the solution, then the flask was flushed with argon and sealed with a rubber septum. The reaction mixture was stirred at room temperature, protected from light. After 2 days 200 ml methylene chloride and 5% ammonia solution was added, and the solution was extracted 5 times with water, then the organic phase was dried over anhydrous magnesium sulfate. Solvent was removed on a rotary evaporator and the residue was purified on a normal-phase silica gel filled column, using methylene chloride: hexane (1:1) as eluent. Yield: 0.36 g, 34 % monoMICAAc (yellow crystals) and 0.29 g, 26 % diMICAAc (orange crystals).

monoMICAAc

^1^H NMR (360 MHz, CDCl_3_) δ= 8.46 (s, 1H), 8.06 (s, 1H), 7.87 (d, *J* = 8.7 Hz, 1H), 7.71 (d, *J* = 9.4 Hz, 1H), 7.28 (dd, *J* = 8.8, 1.7 Hz, 1H), 7.00 – 6.90 (m, 2H), 4.49 (d, *J* = 4.0 Hz, 1H), 3.03 (d, *J* = 5.1 Hz, 4H) ppm.

^13^C NMR (95 MHz, CDCl_3_) δ = 165.44 (C_NC_), 152.69 (C_3_), 151.27 (C_5a,10a_), 148.34 (C_6_), 135.45 (C_9_), 130.14 (C_8_), 129.20 (C_1_), 126.07 (C_7_), 124.10 (C_8a_), 122.56 (C_9a_), 121.70 (C_5_), 120.91 (C_2_), 100.77 (C_4_), 30.34 (C_CH3_) ppm.

ESI-TOF MS (m/z): calculated m/z of [C_15_H_11_N_3_ +H]^+^ adduct ion is 234.103; found; 234.103.

diMICAAc

^1^H NMR (400 MHz, CDCl_3_) δ= 8.48 (s, 1H), 8.05 (s, 1H), 7.86 (d, J = 8.7 Hz, 1H), 7.80 (d, J = 9.4 Hz, 1H), 7.31 (dd, J = 9.4, 2.3 Hz, 1H), 7.26 (d, J = 6.3 Hz, 1H), 7.07 (s, 1H), 3.19 (s, 6H) ppm.

^13^C NMR (101 MHz, CDCl_3_) δ= 165.39 (C_NC_), 152.08 (C_3,5a,10a_), 148.43 (C_6_), 135.40 (C_9_), 130.14 (C_8_), 129.16 (C_1_), 125.95 (C_7_), 124.00 (C_8a_), 121.67 (C_9a_), 120.66 (C_5_), 118.73 (C_2_), 103.28 (C_4_), 40.36 (C_CH3_) ppm.

ESI-TOF MS (m/z): calculated m/z of [C_16_H_13_N_3_ +H]^+^ adduct ion is 248.118; found; 248.117.

### NMR

^1^H and ^13^C-NMR spectra were recorded in CDCl_3_ and DMSO-d_6_ at 25 ºC on a Bruker Avance DRX-400 and a Bruker AM 360 spectrometer at 400 MHz and 360 MHz, respectively with tetramethylsilane as the internal standard.

# Chapter I. 3-amino-6-isocyanoacridine (ICAAc)


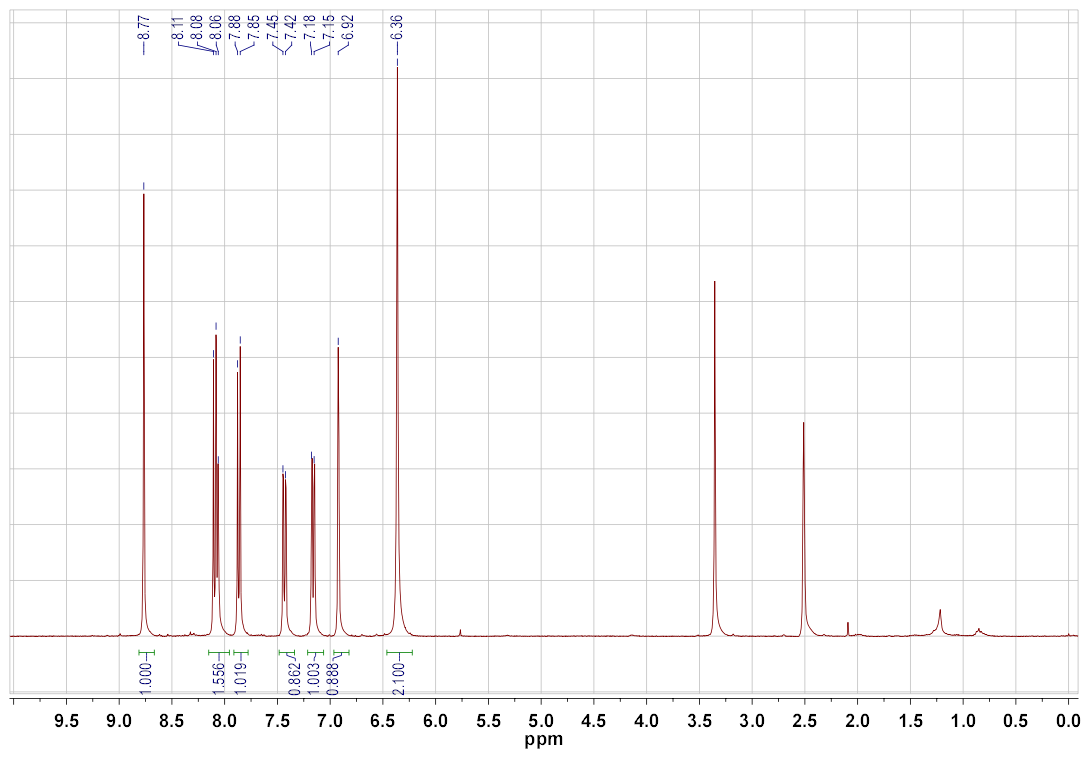


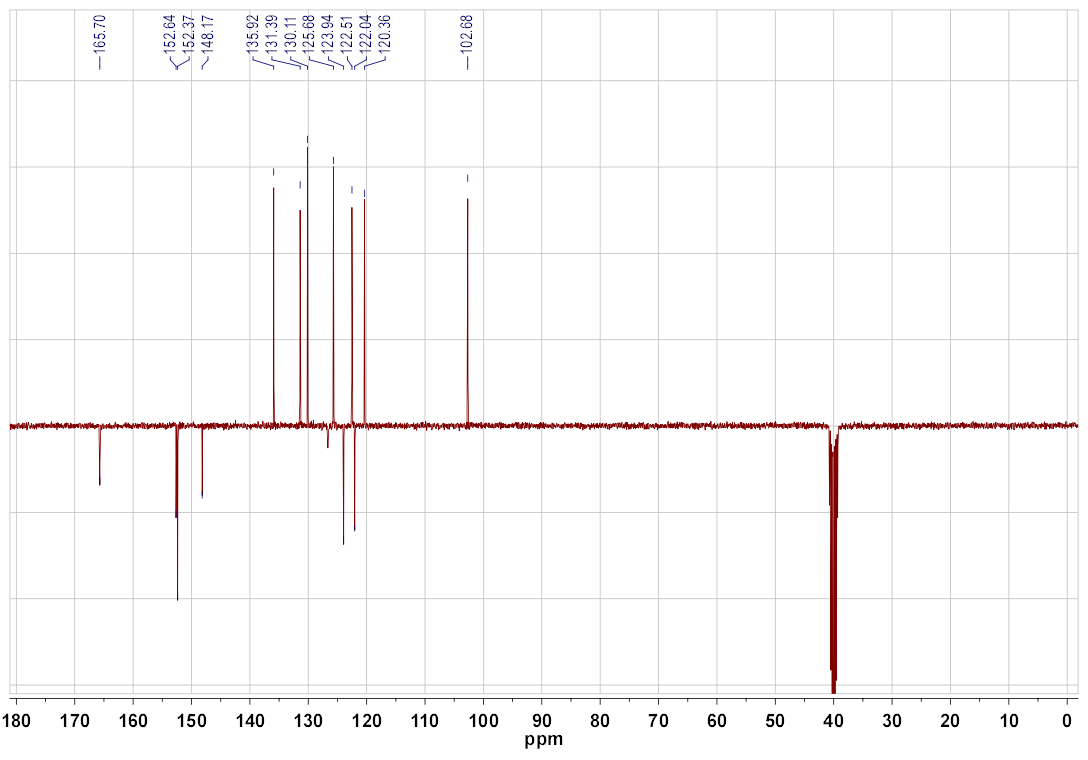


1. ^1^H-NMR (top) and ^13^C-NMR (bottom) spectra of ICAAc

recorded at 20 °C in DMSO-d6


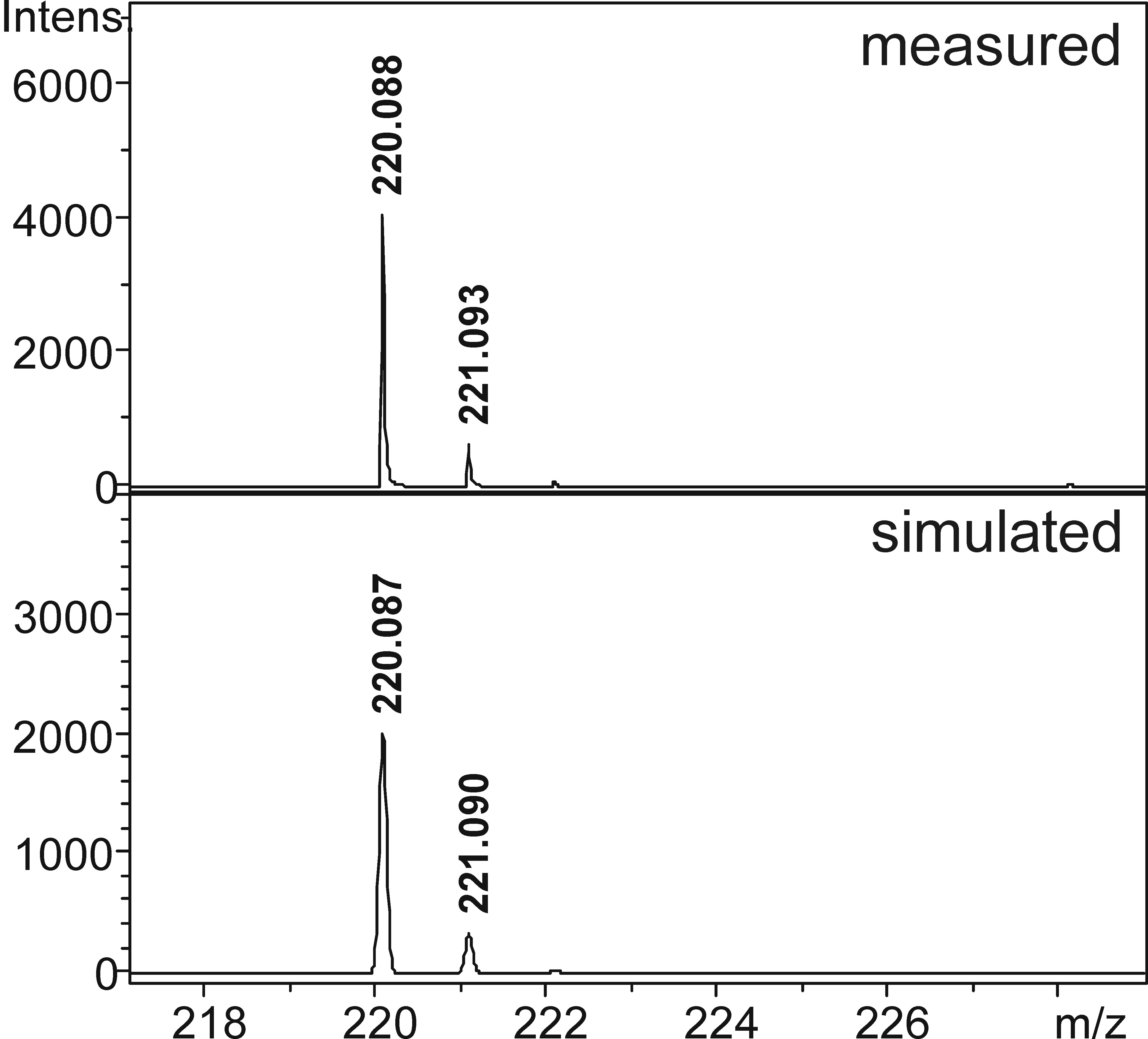


1. The measured and calculated ESI-MS spectra of ICAAc

### Experimental conditions of Electrospray Quadrupole Time-of-Flight MS/MS (ESI-Q-TOF)

The MS and MS/MS measurements were carried out by a MicroTOF-Q type Qq-TOF MS instrument (Bruker Daltonik, Bremen, Germany). The instrument was equipped with an electrospray ion source where the spray voltage was 4 kV. N_2_ was utilized as drying gas. The temperature of the drying gas was 200 °C and the flow rate was 4.0 L/min. For the tandem MS measurements collision gas was nitrogen. The precursor ions for MS/MS were selected with an isolation width of 4 *m/z* units. The mass spectra were recorded by means of a digitizer at a sampling rate of 2 GHz. The mass spectra were calibrated externally using the exact masses of cluster ions of sodium trifluoracetate [(NaTFA)_n_+Na]^+^ generated under electrospray condition. The spectra were evaluated with the DataAnalysis 3.4 software from Bruker. The samples were introduced by a syringe pump (Cole-Parmer Ins. Co., Vernon Hills, IL, USA) at a flow rate of 10 μL/min. For the mass spectrometric measurements the samples were dissolved in methanol in the concentration of 0.1 mM.


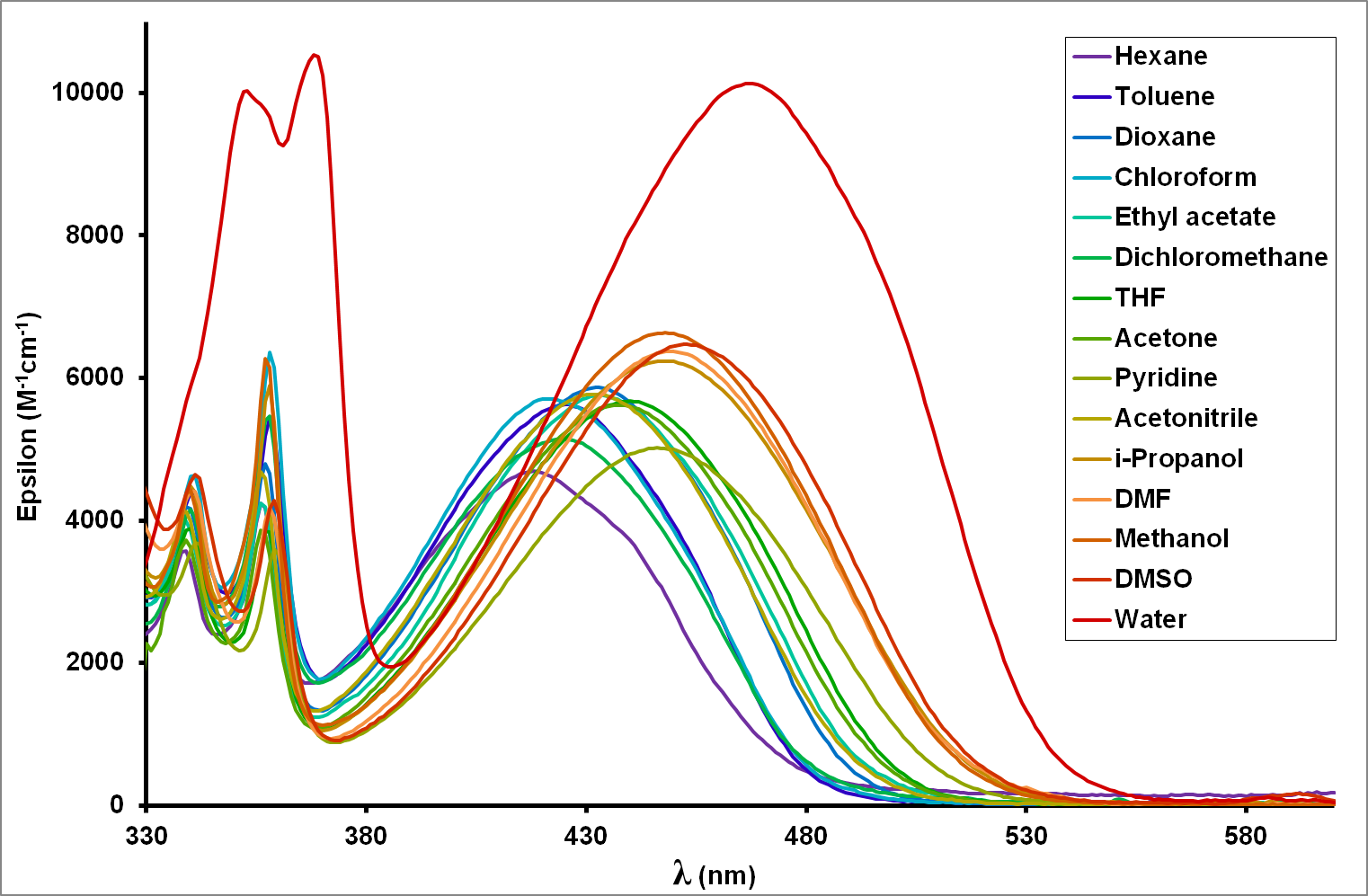


1. UV-Vis absorbance spectrum of of ICAAc recorded in different solvents

(20 °C, c=5.2*10^-6^ M)


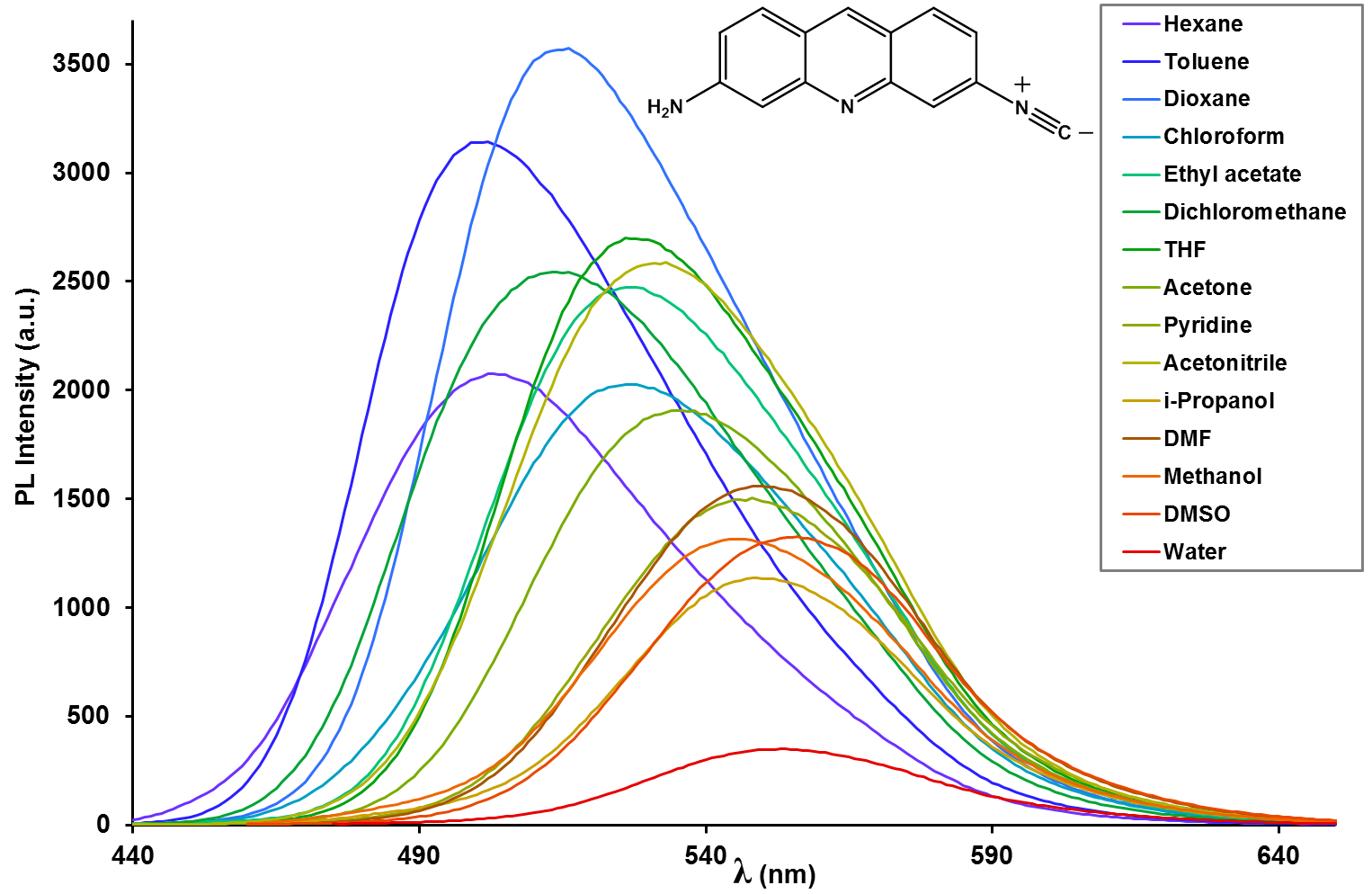


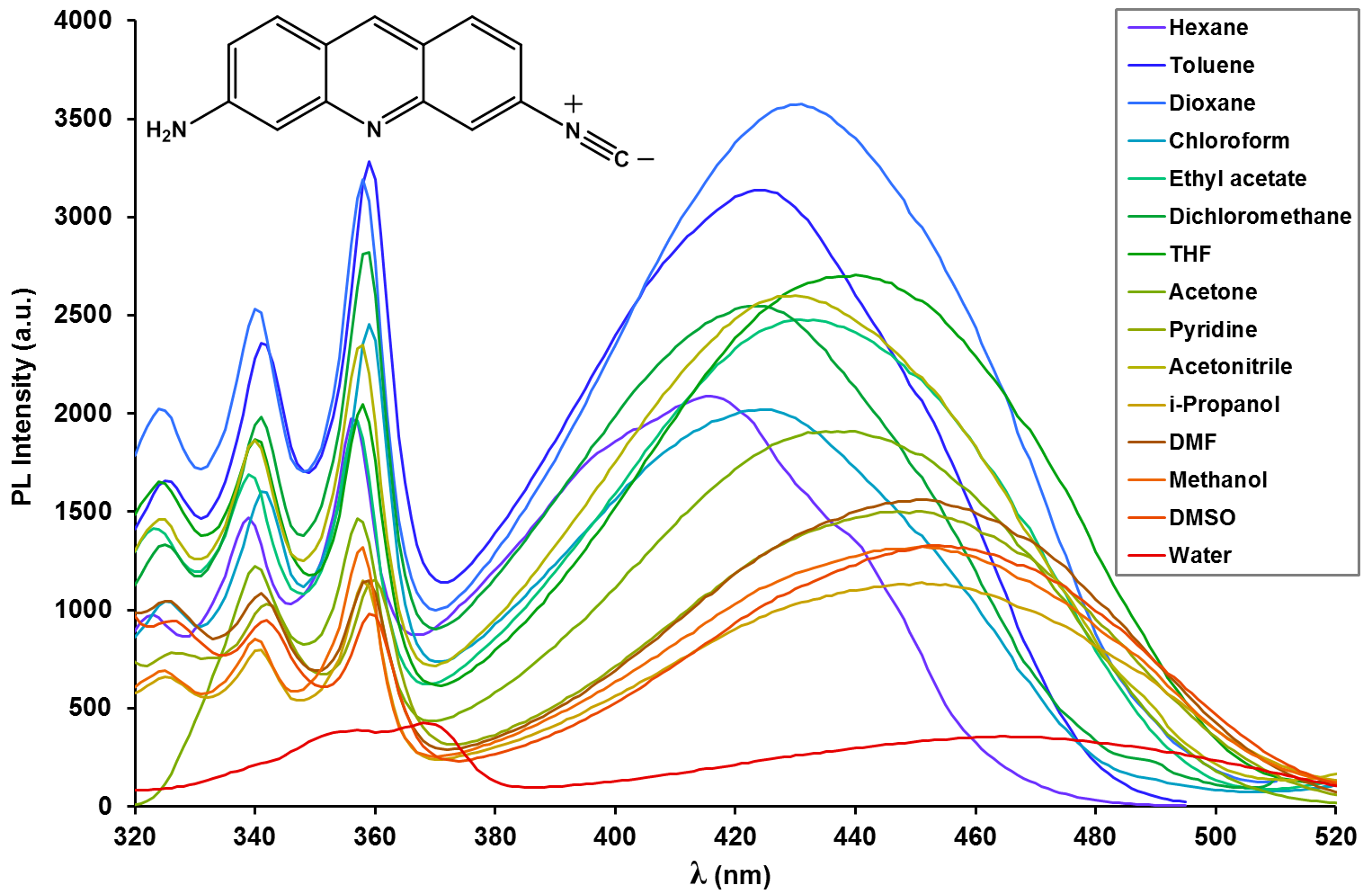


1. Fluorescence emission (top) and excitation (bottom) spectra of ICAAc recorded in different solvents

(20 °C, c=5.2*10^-6^ M)

1. Emission (λ_em_), excitation (λ_ex_) maxima, Stokes shift (Δν), molar absorbance (ε) and quantum yield (Φ_F_) of ICAAc in different solvents

# Chapter II. 3-N-methylamino-6-isocyanoacridine (monoMICAAc)


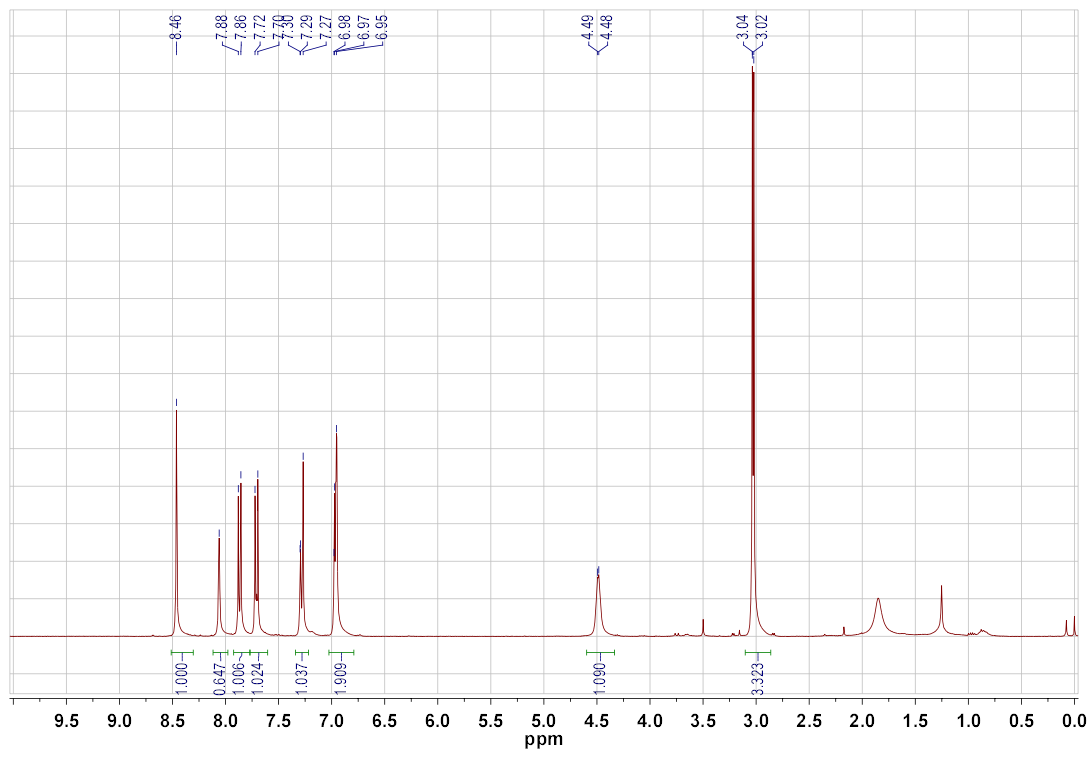


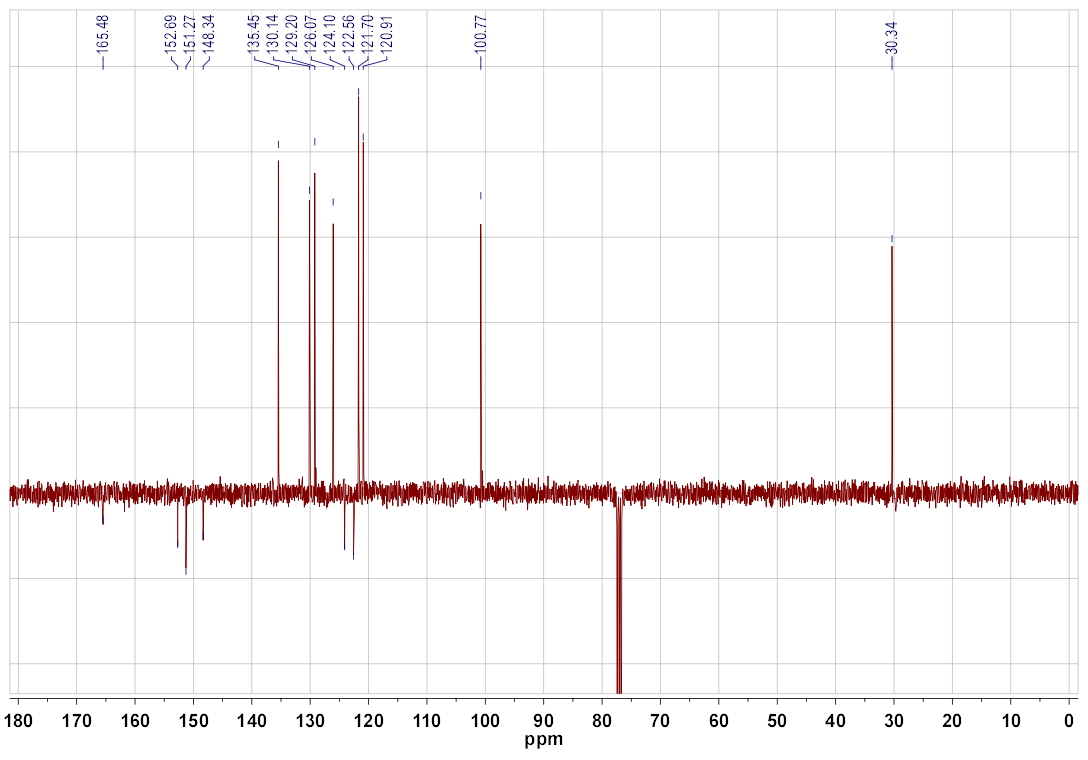


1. ^1^H-NMR (top) and ^13^C-NMR (bottom) spectra of monoMICAAc

recorded at 20 °C in CDCl_3_


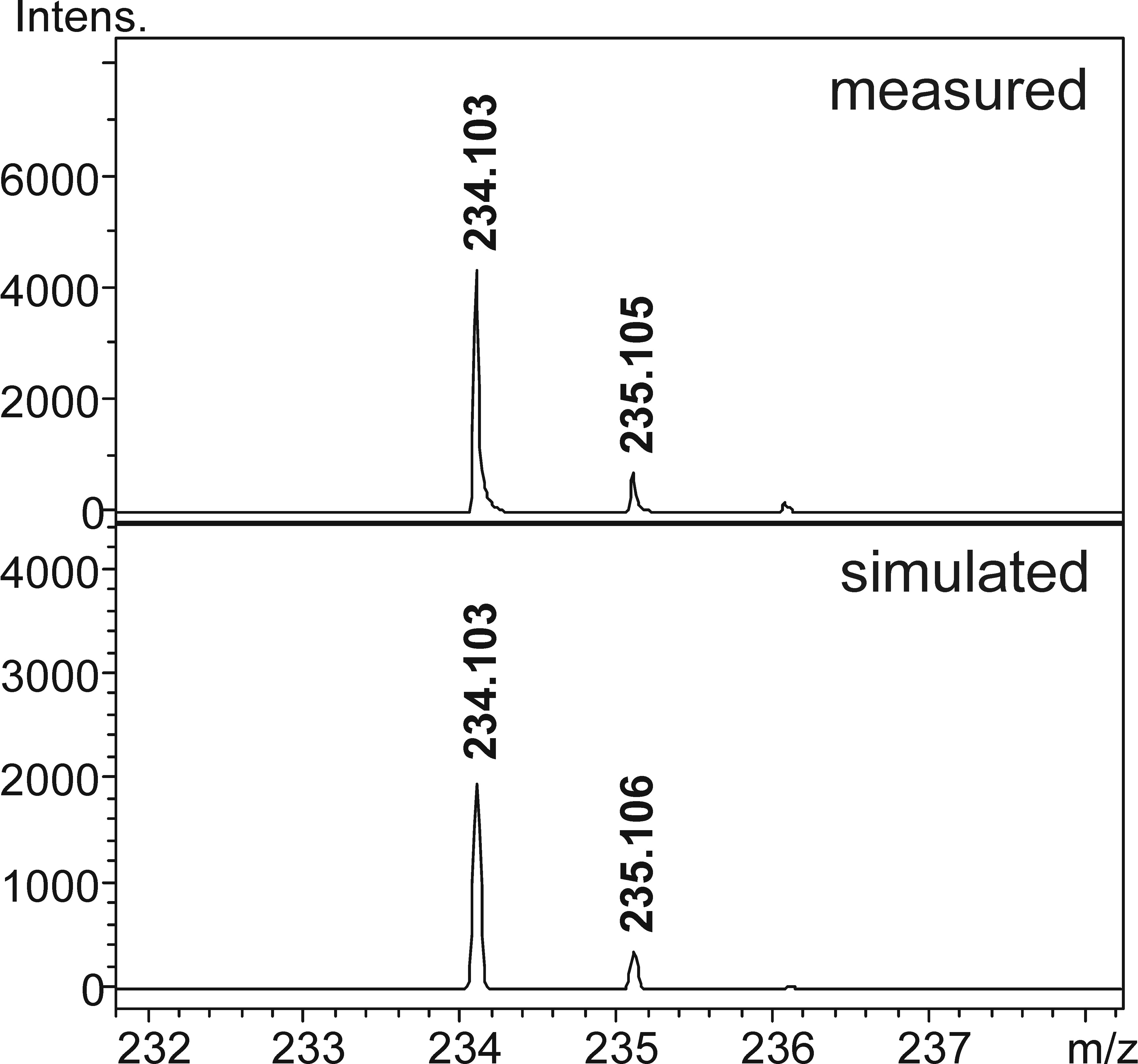


1. The measured and calculated ESI-MS spectra of monoMICAAc


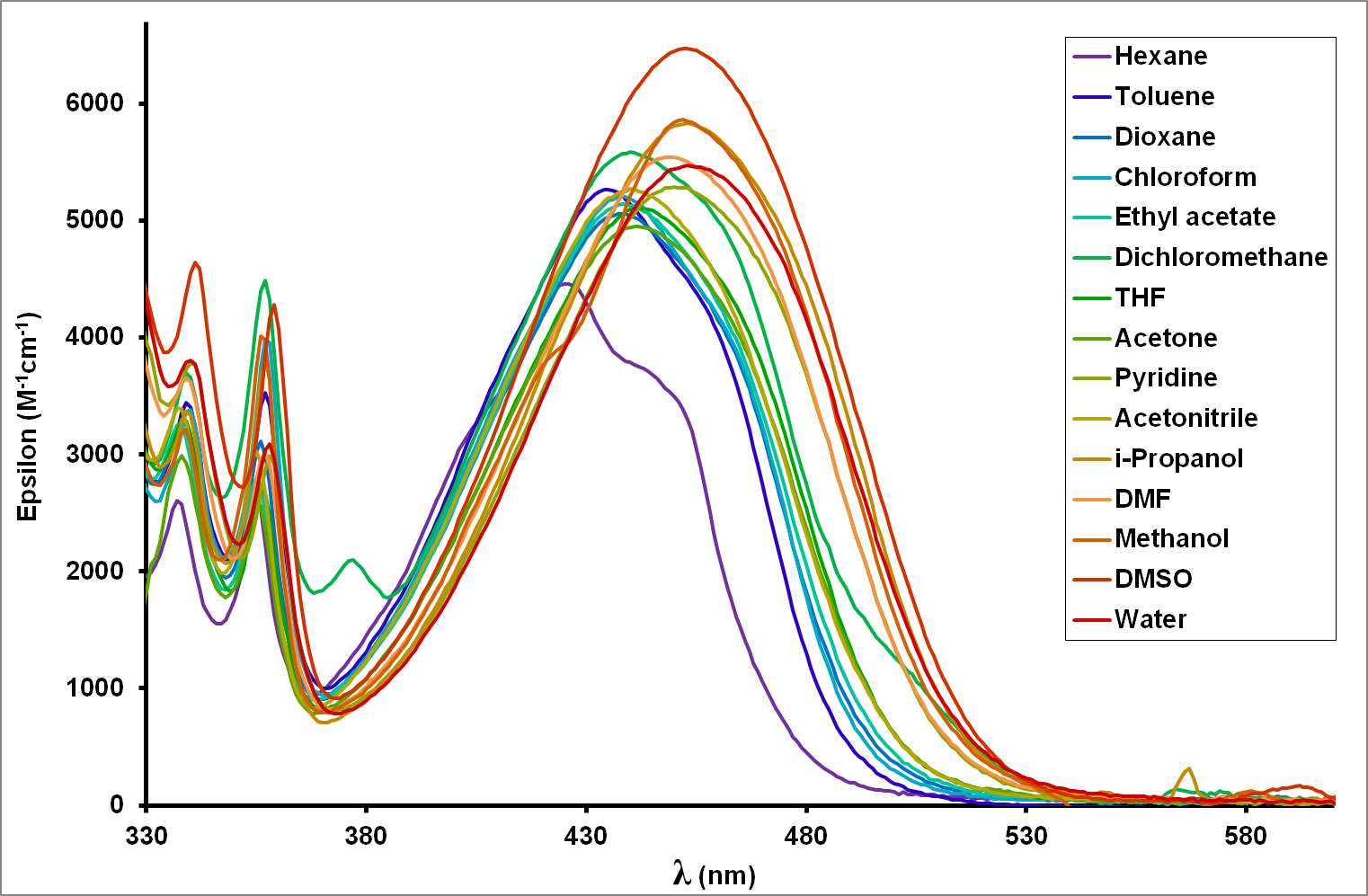


1. UV-Vis absorbance spectrum of monoMICAAc recorded in different solvents

(20 °C, c=4.1*10^-6^ M)


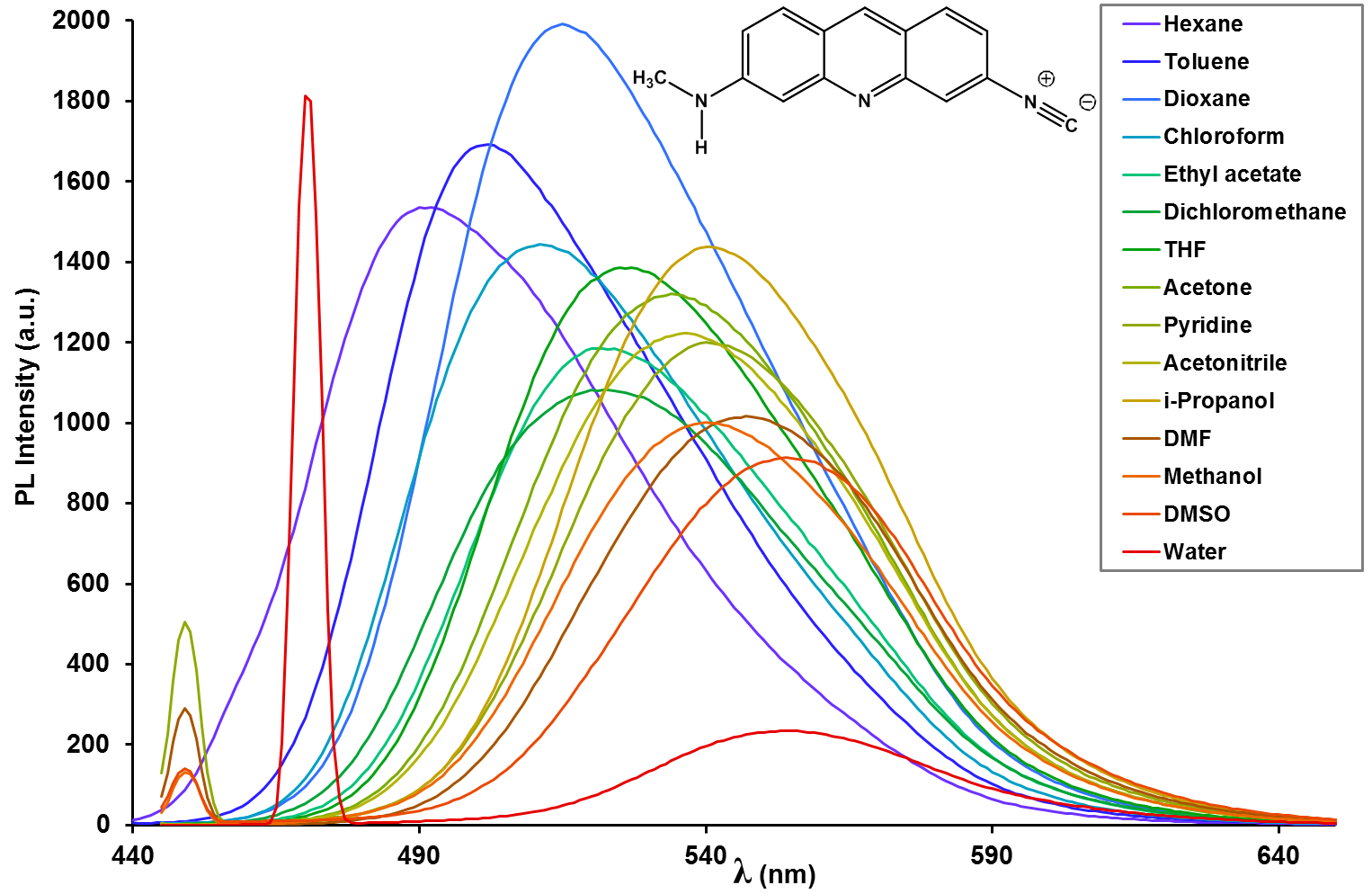


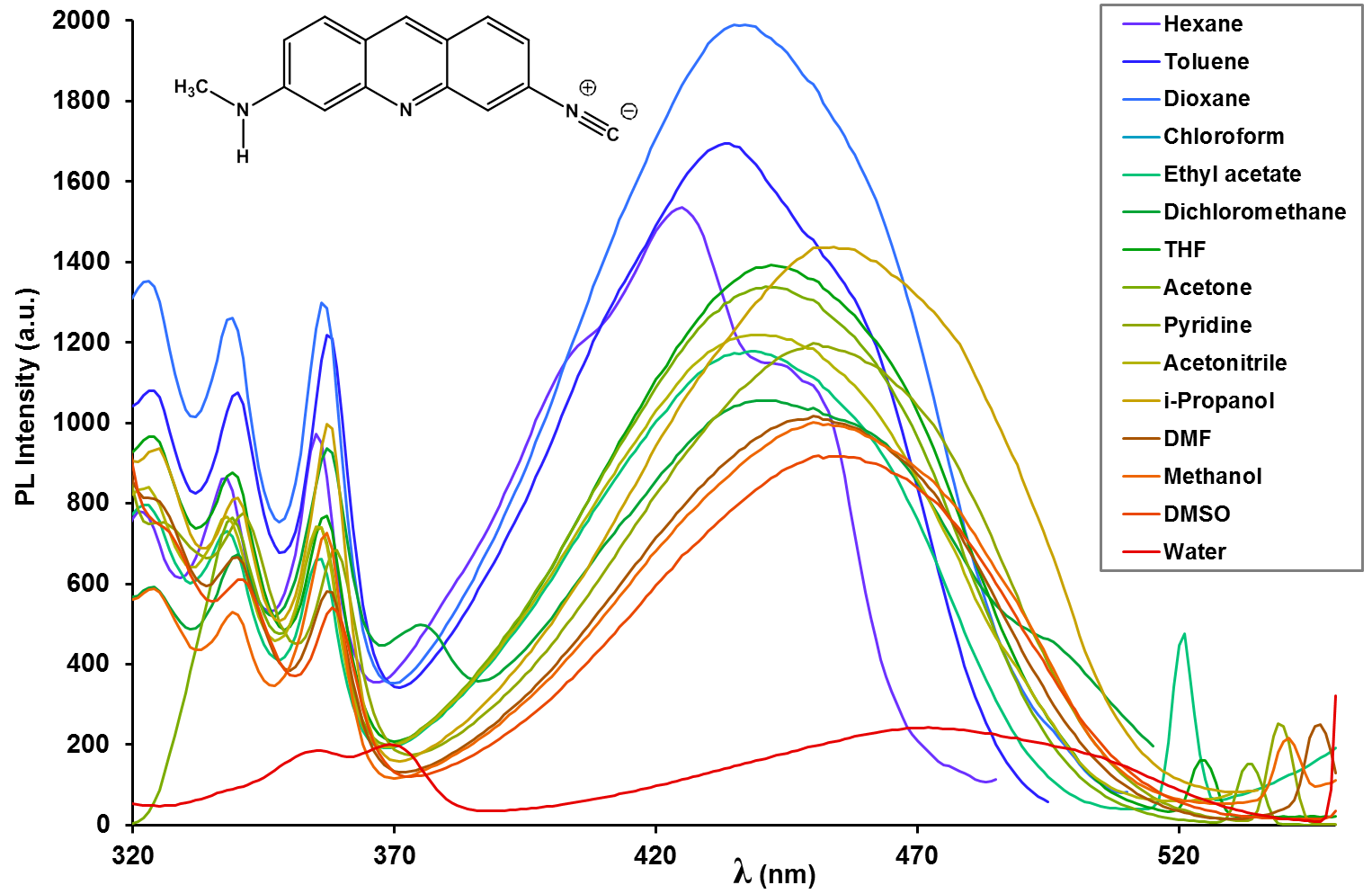


1. Fluorescence emission (top) and excitation (bottom) spectra of monoMICAAc recorded in different solvents

(20 °C, c=4.1*10^-6^ M)

1. Emission (λ_em_), excitation (λ_ex_) maxima, Stokes shift (Δν), molar absorbance (ε) and quantum yield (Φ_F_) of monoMICAAc in different solvents

# Chapter III. 3-N,N-dimethylamino-6-isocyanoacridine (diMICAAc)


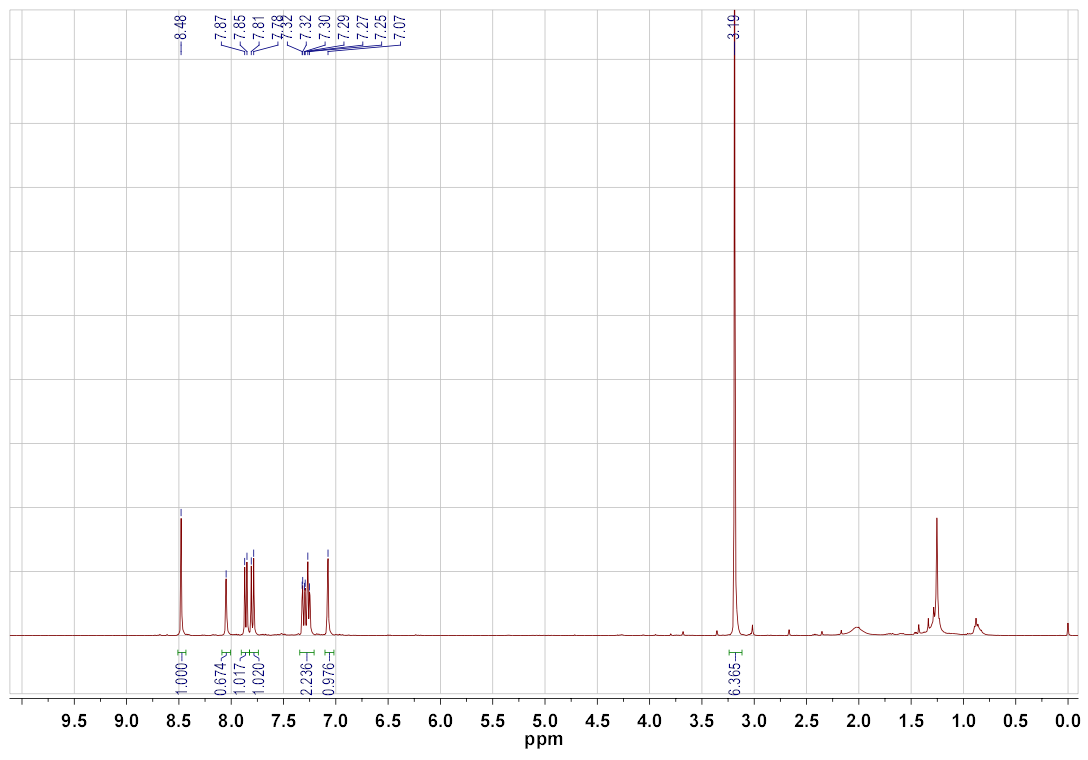


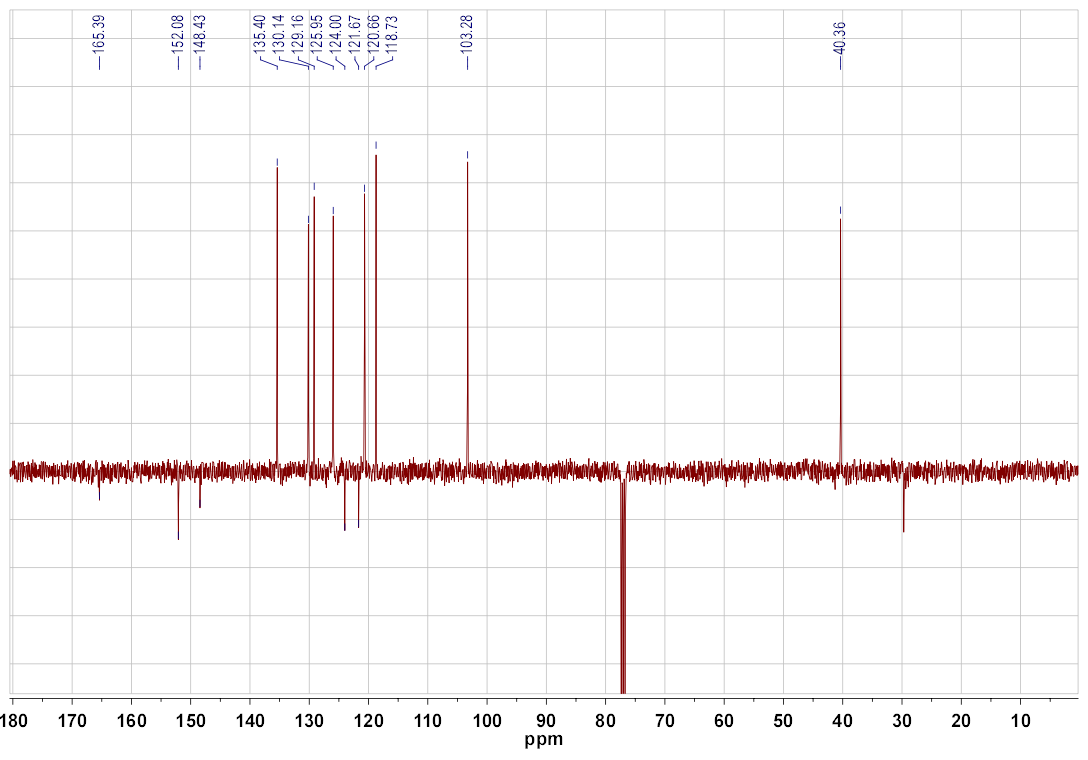


1. ^1^H-NMR (top) and ^13^C-NMR (bottom) spectra of diMICAAc

recorded at 20 °C in CDCl_3_


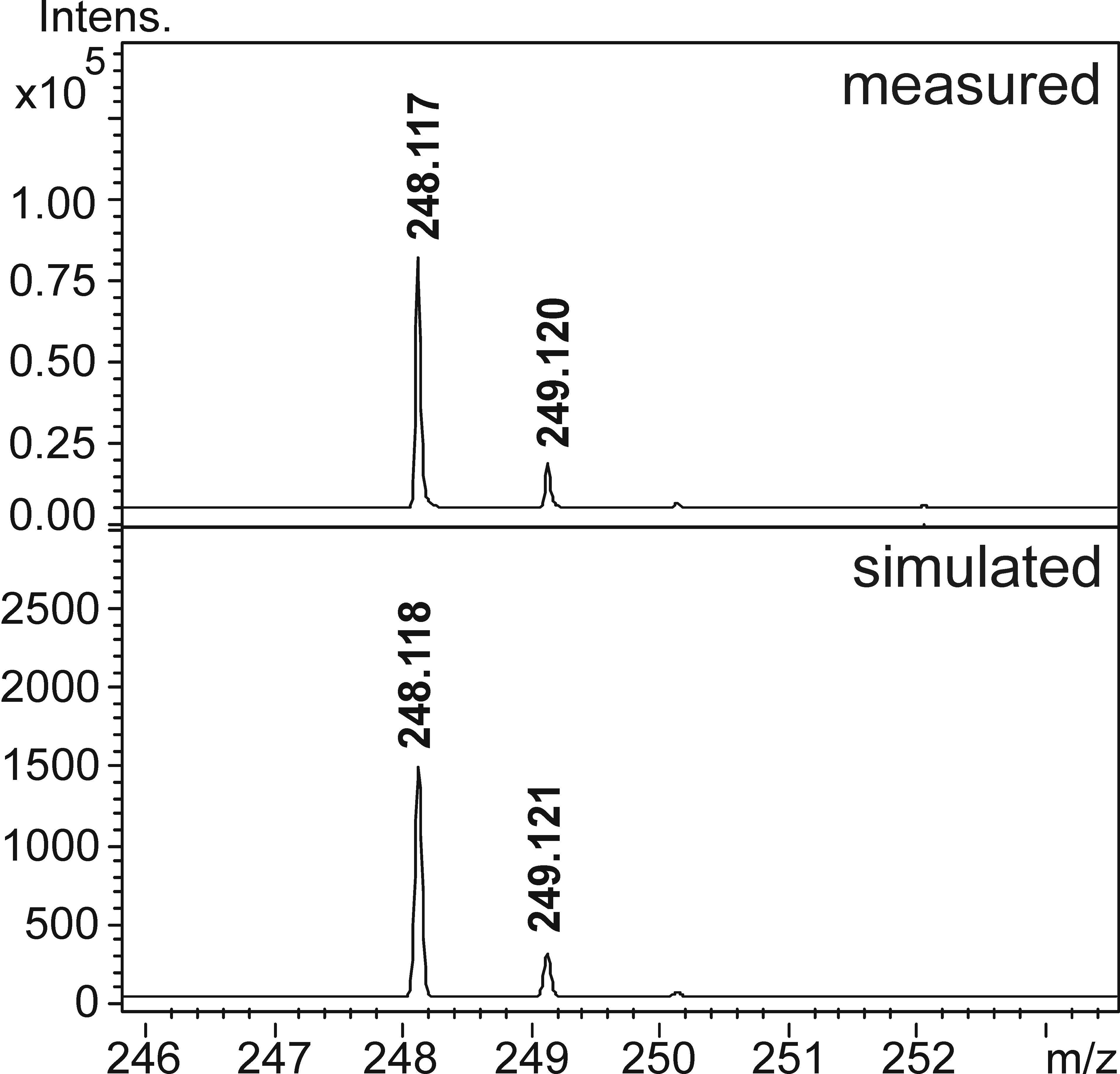


1. The measured and calculated ESI-MS spectra of diMICAAc


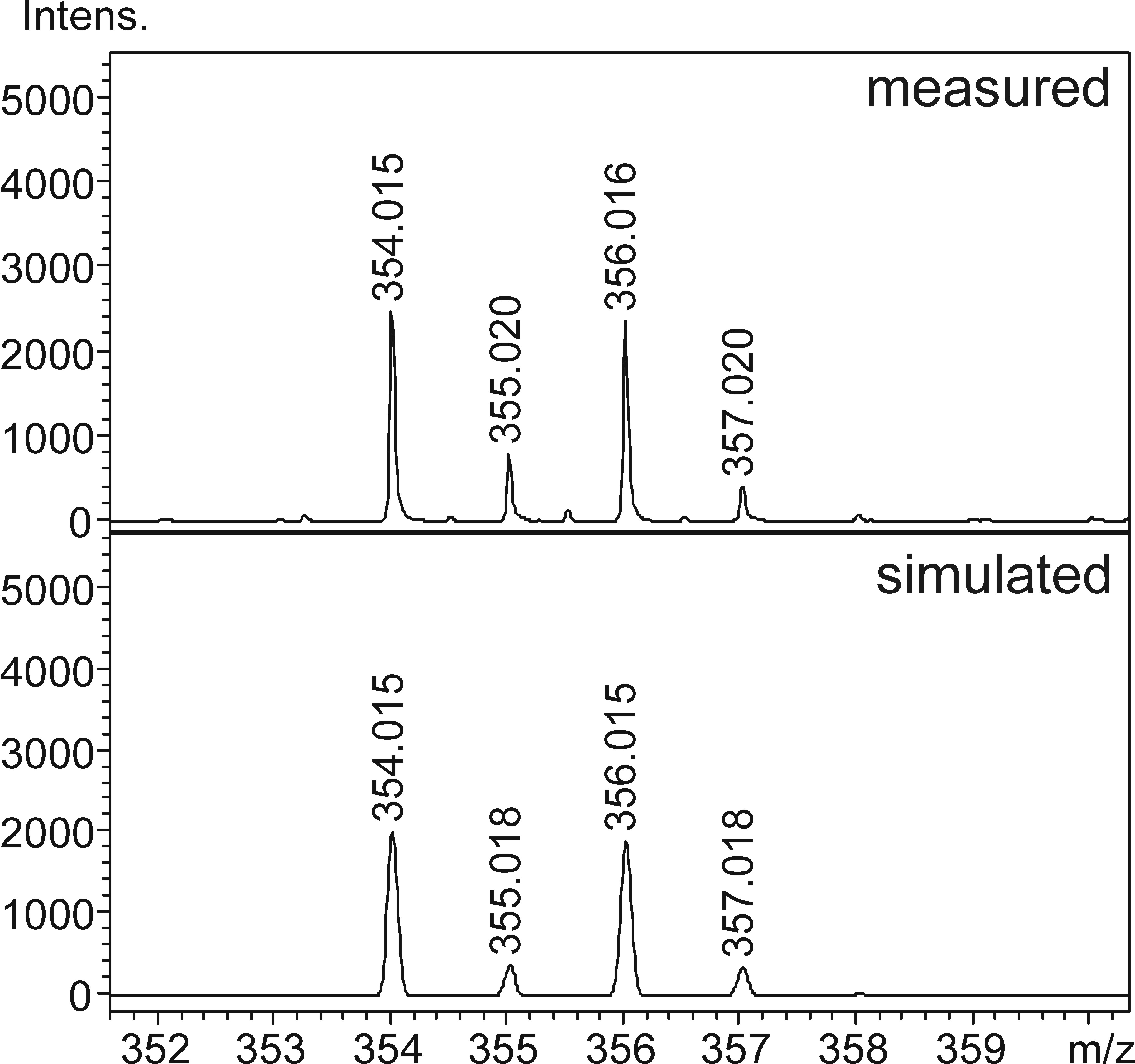


1. The measured and calculated ESI-MS spectra of [Ag(diMICAAc)]^+^


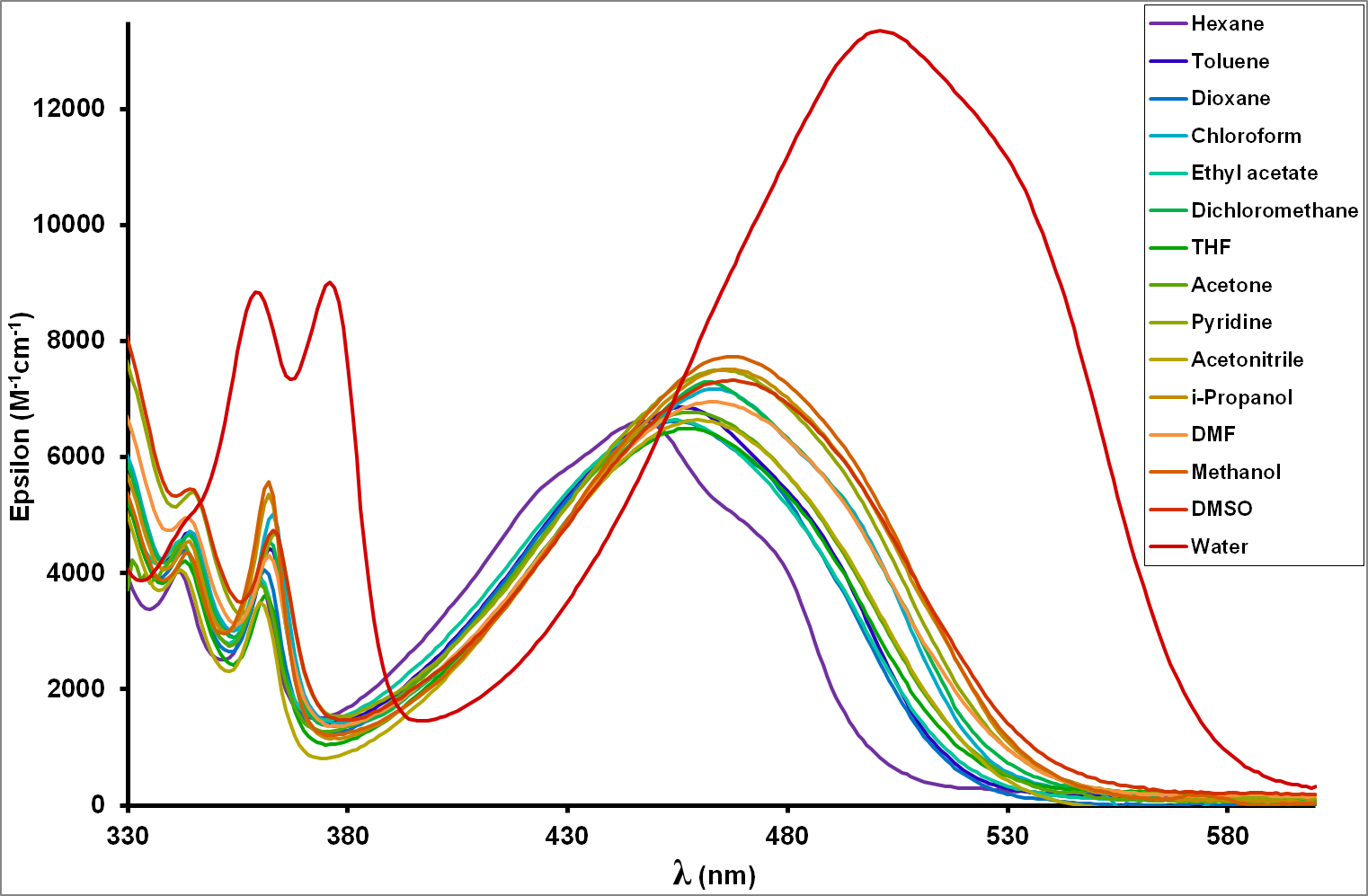


1. UV-Vis absorbance spectrum of diMICAAc recorded in different solvents

(20 °C, c=4.6*10^-6^ M)


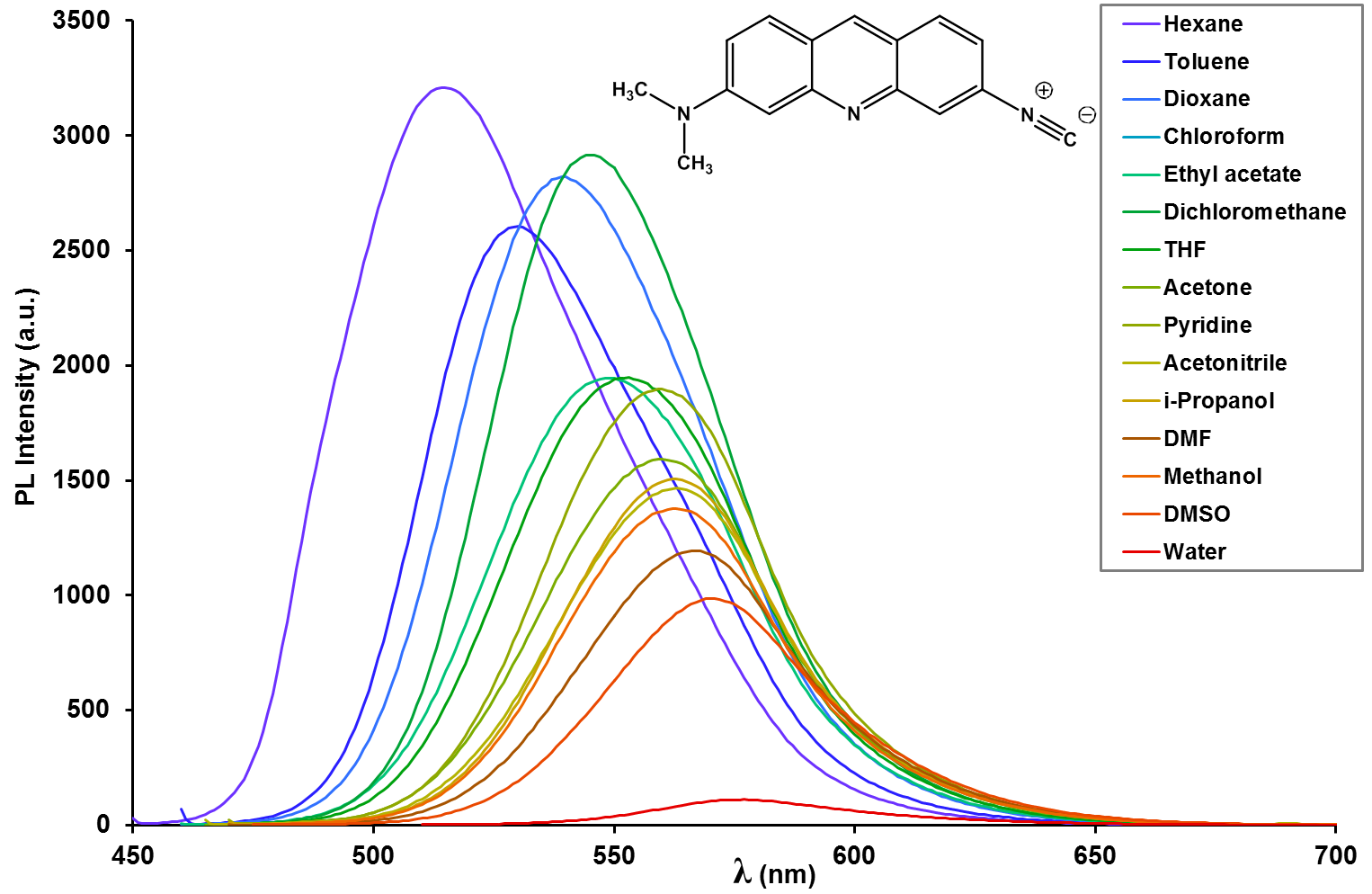


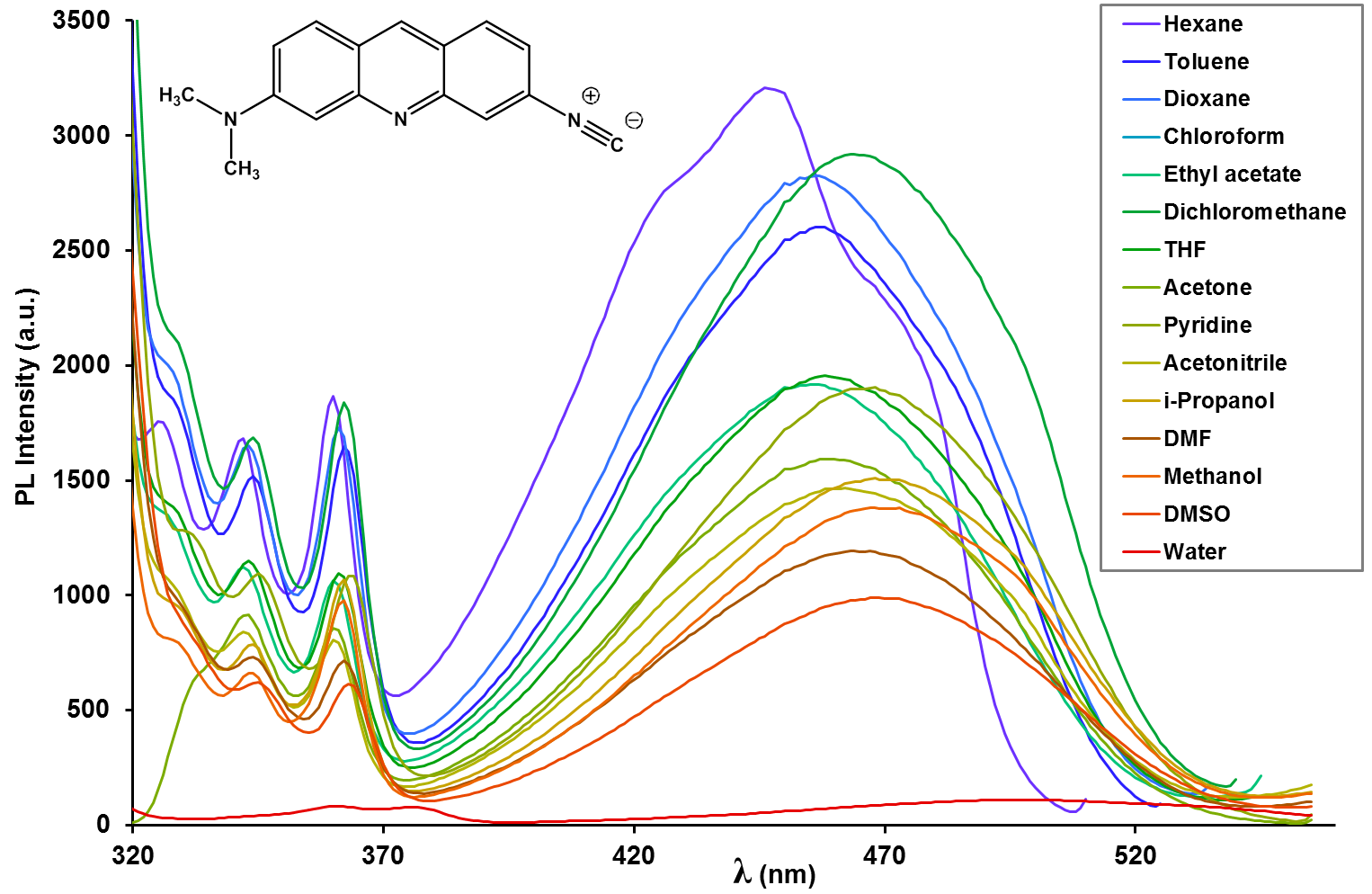


1. Fluorescence emission (top) and excitation (bottom) spectra of diMICAAc recorded in different solvents

(20 °C, c=4.6*10^-6^ M)

1. Emission (λ_em_), excitation (λ_ex_) maxima, Stokes shift (Δν), molar absorbance (ε) and quantum yield (Φ_F_) of diMICAAc in different solvents

| **Solvent** | **Polarity** | **λ_em,max_** | **λ_ex,max_** | **Stokes shift** | **ε** | **φ_F_** |
| --- | --- | --- | --- | --- | --- | --- |
|  | **Index** | **(nm)** | **(nm)** | **(cm^-1^)** | **(M^-1^)** | **(%)** |
| Hexane | 0.0 | 514 | 447 | 2916 | 6616 | 30 |
| Toluene | 2.4 | 529 | 457 | 2978 | 6862 | 24 |
| Dichloromethane | 3.1 | 545 | 464 | 3203 | 7296 | 23 |
| i-propanol | 3.9 | 563 | 469 | 3560 | 7516 | 11 |
| THF | 4.0 | 552 | 458 | 3718 | 6493 | 18 |
| Chloroform | 4.1 | 535 | 464 | 2860 | 7172 | 26 |
| Ethyl acetate | 4.4 | 549 | 456 | 3715 | 6644 | 17 |
| Dioxane | 4.8 | 539 | 456 | 3377 | 6612 | 26 |
| Acetone | 5.1 | 560 | 458 | 3977 | 6769 | 13 |
| Methanol | 5.1 | 562 | 468 | 3574 | 7727 | 9.5 |
| Pyridine | 5.3 | 560 | 468 | 3510 | 7502 | 15 |
| Acetonitrile | 5.8 | 563 | 461 | 3930 | 6640 | 12 |
| Dimethyl formamide | 6.4 | 567 | 465 | 3869 | 6959 | 9.9 |
| Dimethyl sulfoxide | 7.2 | 570 | 468 | 3824 | 7323 | 8.0 |
| Water | 9.0 | 576 | 497 | 2760 | 13344 | 0.4 |

# Chapter IV. Environment-sensitivity of the fluorophores

# Lippert-Mataga equation

The Lippert-Mataga plot was constructed (Fig. 4.a.) by using the relation:

$\Delta\overline{v}_{ss}=\frac{2(\mu_{e}-\mu_{g})^{2}}{4\pi\varepsilon_{\text{o}}\text{hca}^{3}}\Delta\text{f}+\text{const}$ (S1)

where $\Delta\overline{v}_{ss}$ (in cm^-1^) is the Stokes shift, μ_e_ and μ_g_ are the dipole moments of the excited and the ground states, respectively. h, c and ε_o_ are the Planck’s constant, speed of the light in vacuum and the permittivity of the vacuum, respectively. a is the radius of a spherical cavity in which the fluorophore molecule resides.

Δf is the orientation polarizability that can be expressed as:

$\Delta\text{f}=\frac{\varepsilon\text{-1}}{2\varepsilon\text{-1}}-\frac{\text{n}^{2}}{\text{2n}^{2}-1}$ (S2)

where ε and n are the dielectric constant and the refractive index of the solvent, respectively.

**
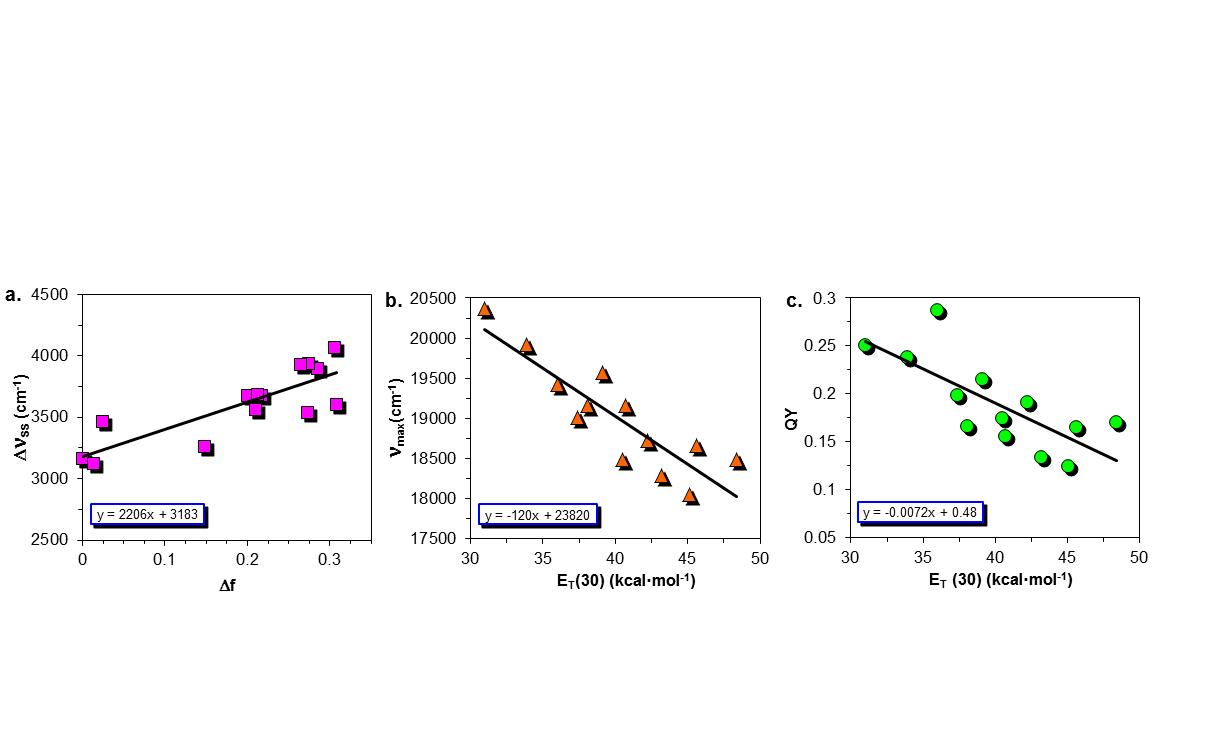
**

1. Demonstration of the variation of the photophysical properties of **monoMICAAc** as a function of solvent polarity: (**a**) Stokes shift *vs* orientation polarizability (Δf) (Lippert-Mataga plot), (**b**) emission maximum *vs* solvent polarity parameter E_T_(30) (**c**) quantum yield *vs* E_T_(30).

| 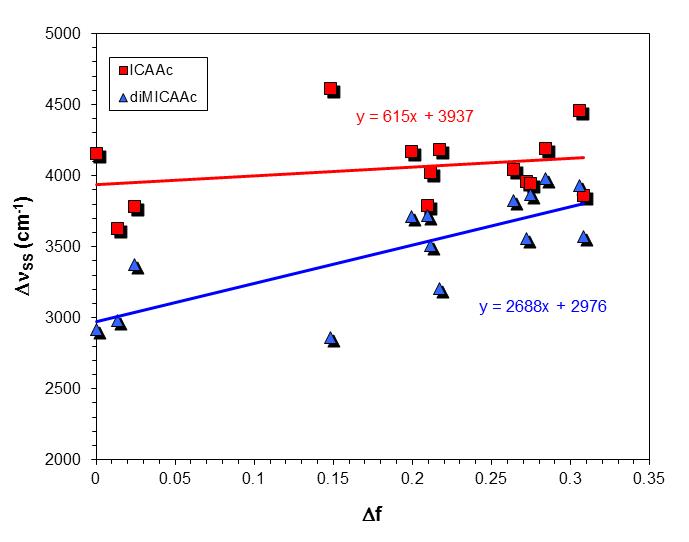 |
| --- |
| 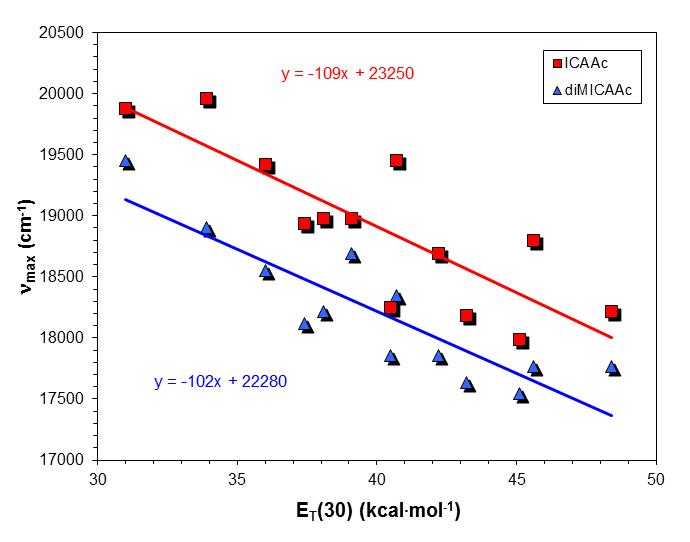 |
| 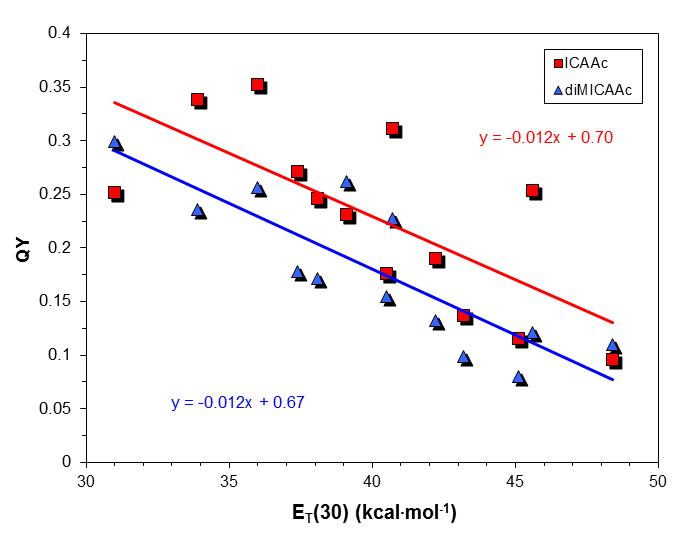 |

1. Demonstration of the variation of the photophysical properties of ICAAc and diMICAAc as a function of solvent polarity

(a) Stokes shift vs orientation polarizability (Lippert Mataga plot), (b) emission maximum vs solvent polarity parameter E_T_(30) (c) Quantum yield vs E_T_(30).
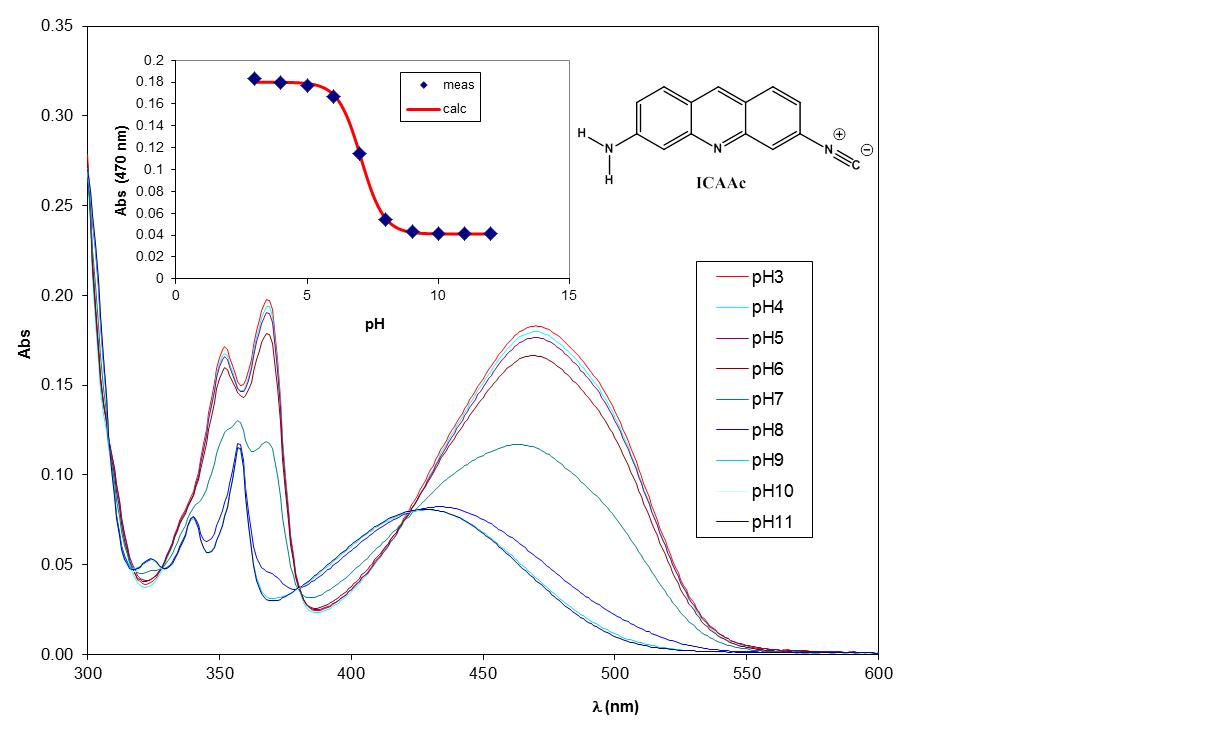


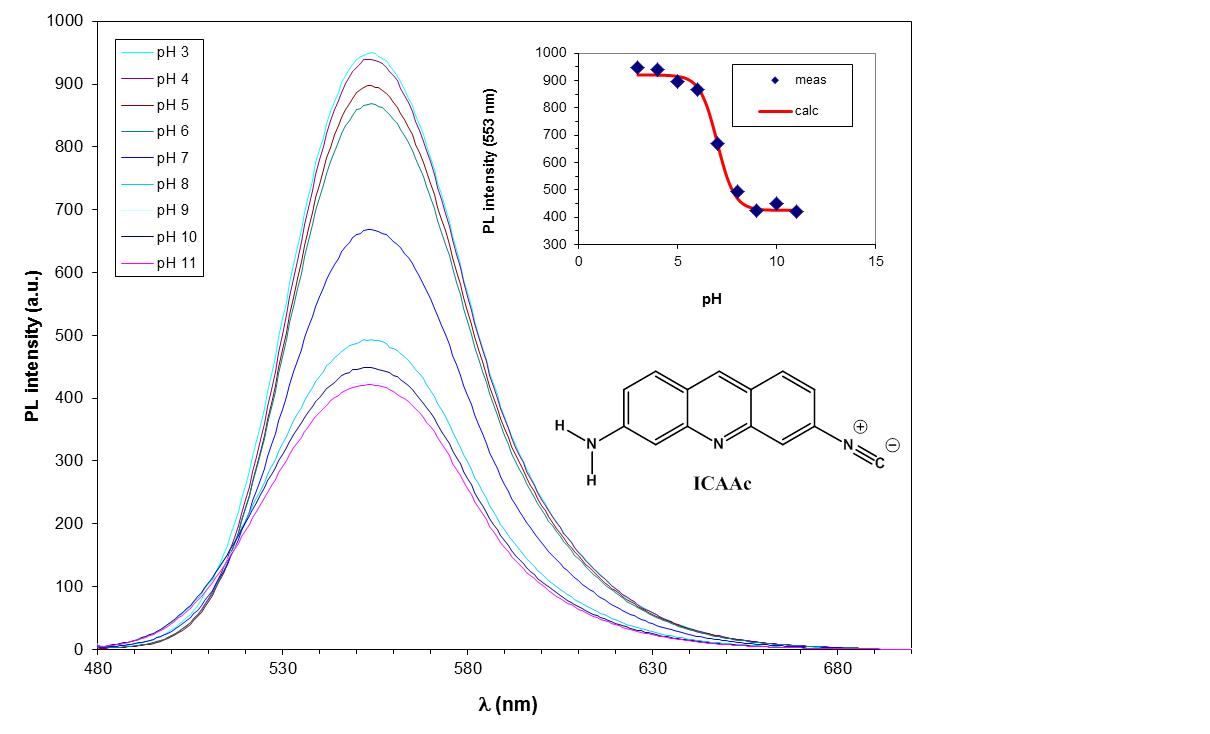


1. Demonstration of the changes in the UV-vis absorption (**top**) and emission (**bottom**) spectrum of **ICAAc** in Britton–Robinson „universal” buffer at different pH

The insets show Eq. 1 fitted (continuous line) on the experimental absorbance or PL intensity data. (T=20 ºC. ([dye] = 4.58×10^-5^ M), V=3.00 cm^3^).


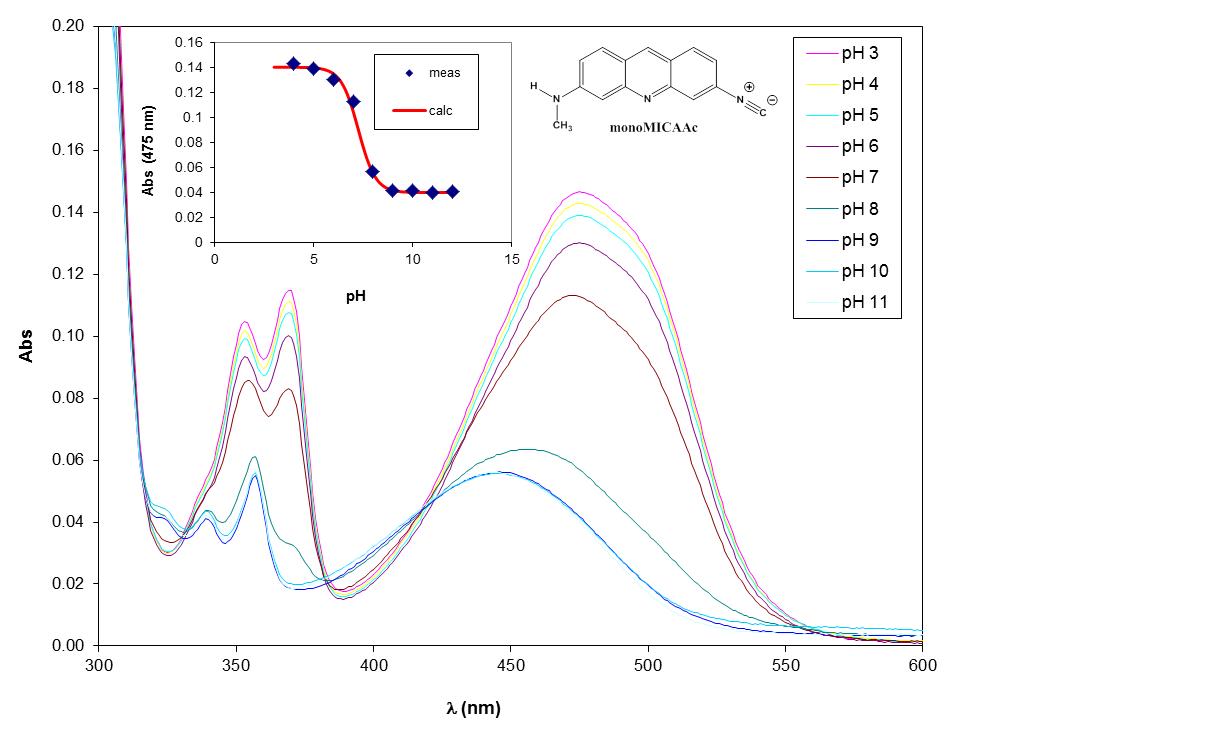


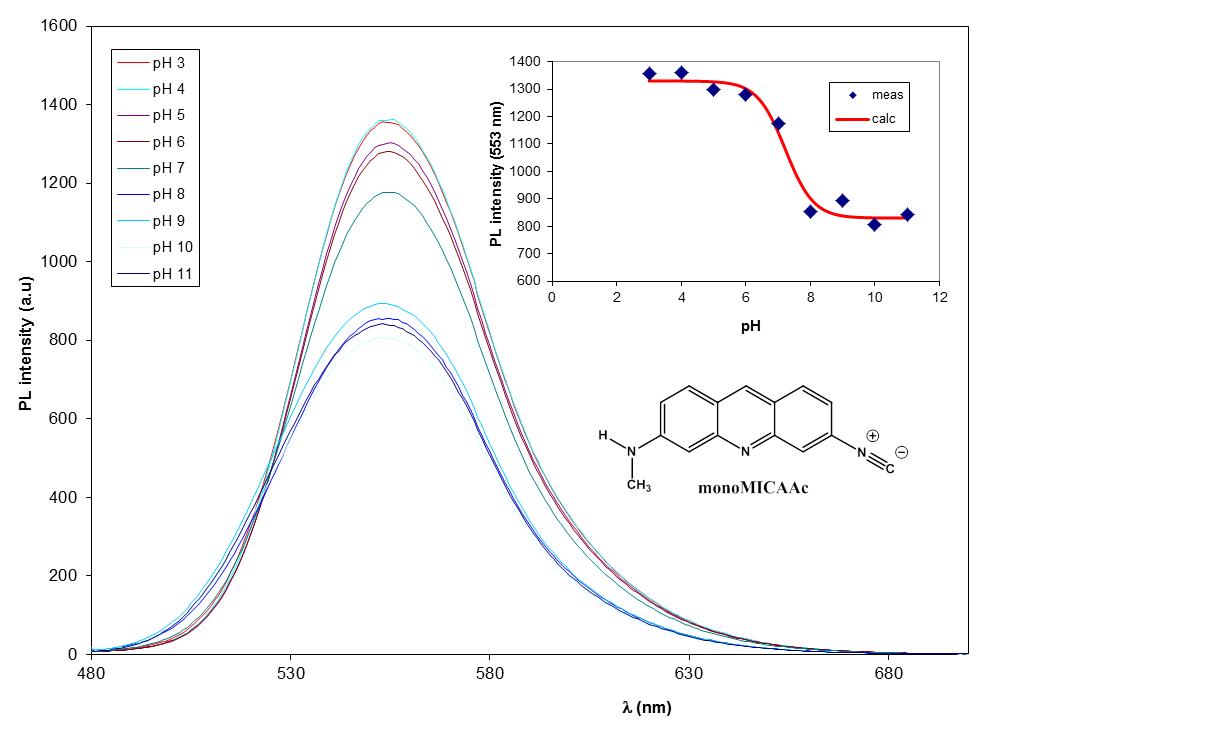


1. Demonstration of the changes in the UV-vis absorption (**top**) and emission (**bottom**) spectrum of **monoMICAAc** in Britton–Robinson „universal” buffer at different pH.

The insets show Eq. 1 fitted (continuous line) on the experimental absorbance or PL intensity data. (T=20 ºC. ([dye] = 4.58×10^-5^ M), V=3.00 cm^3^).


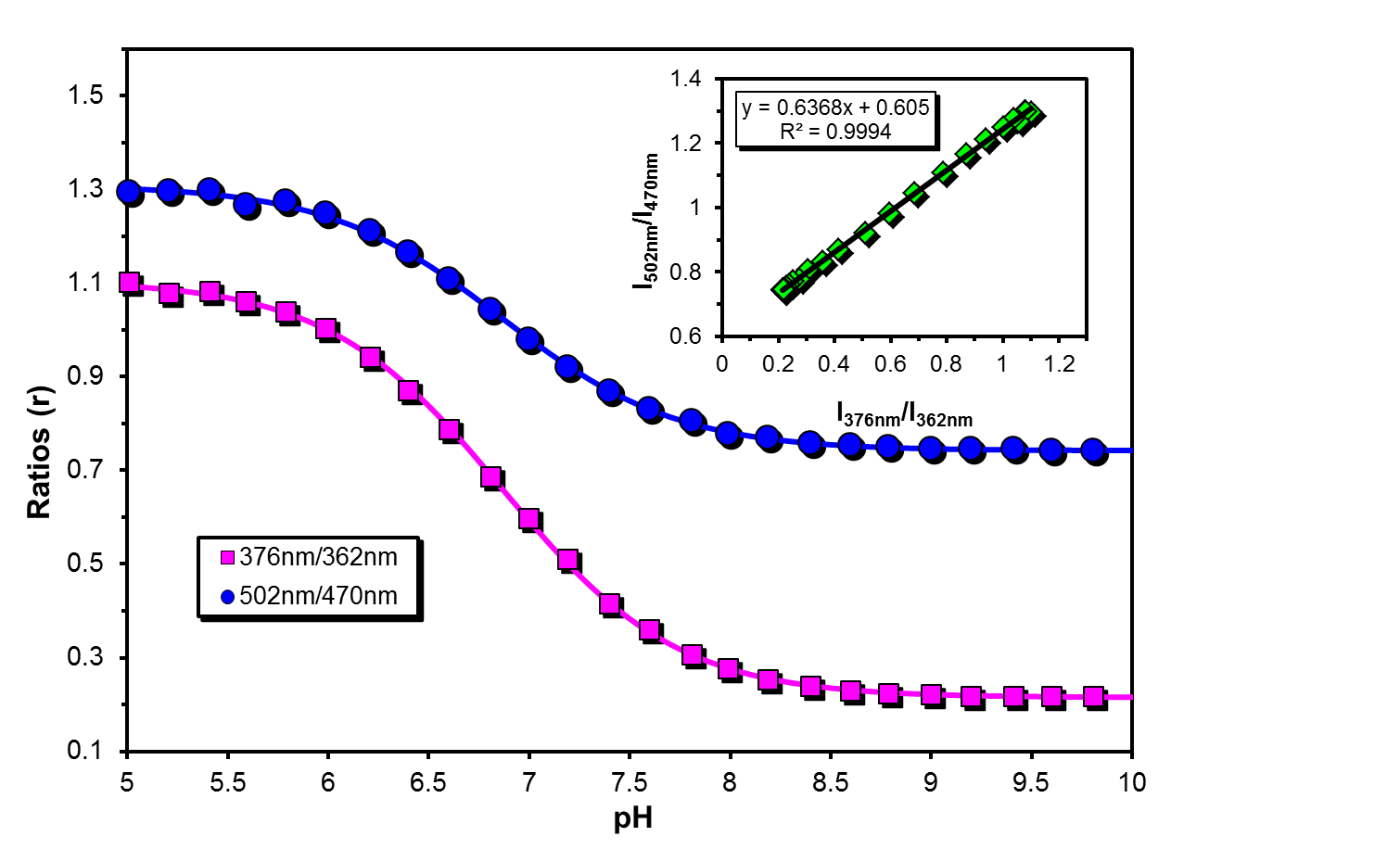


1. Fluorescence (excitation) titration curves of diMICAAc in Britton–Robinson buffer at different pHs

The continuous lines were fitted using eq. 2. The inset shows the correlation between the excitation intensity ratios calculated at the CT and acridine like wavelengths. (T=20 ºC, [dye] = 1.14×10^-5^ M, V=3.00 cm^3^) Parameters used for fitting. (b_1_=7.78x10^-8^, b_2_=1.05x10^-7^ and b_3_= 7.46x10^-1^ for the **I**_502_/**I**_470_ ratio and b_1_=2.65x10^-8^, b_2_=1.23x10^-7^ and b_3_= 9.05x10^-1^ for the **I**_376_/**I**_362_ ratio, respectively).

##
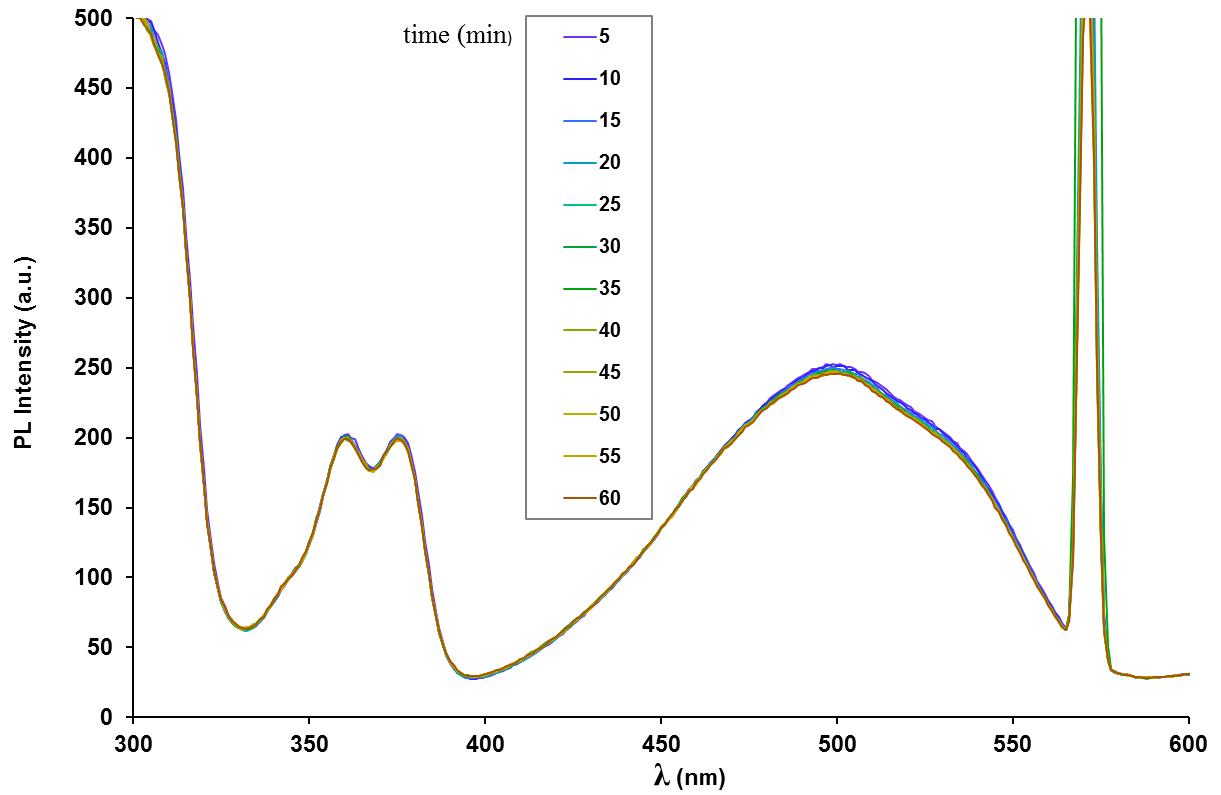


1. Stability test of the fluorescence of diMICAAc in phosphate buffer at pH=6


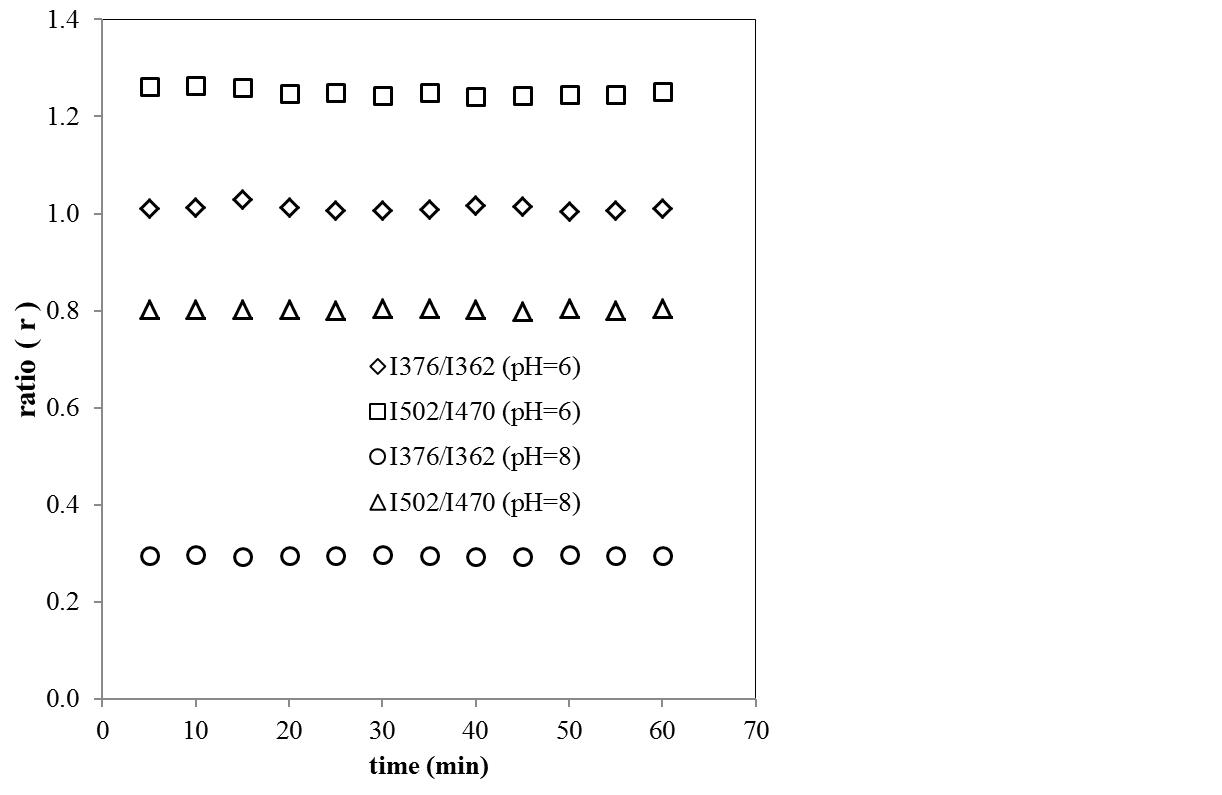


1. Stability test of the fluorescence of diMICAAc in phosphate buffers at pH=6 and 8. Symbols represent the intensity ratios of the excitation peaks extracted from the fluorescence spectra


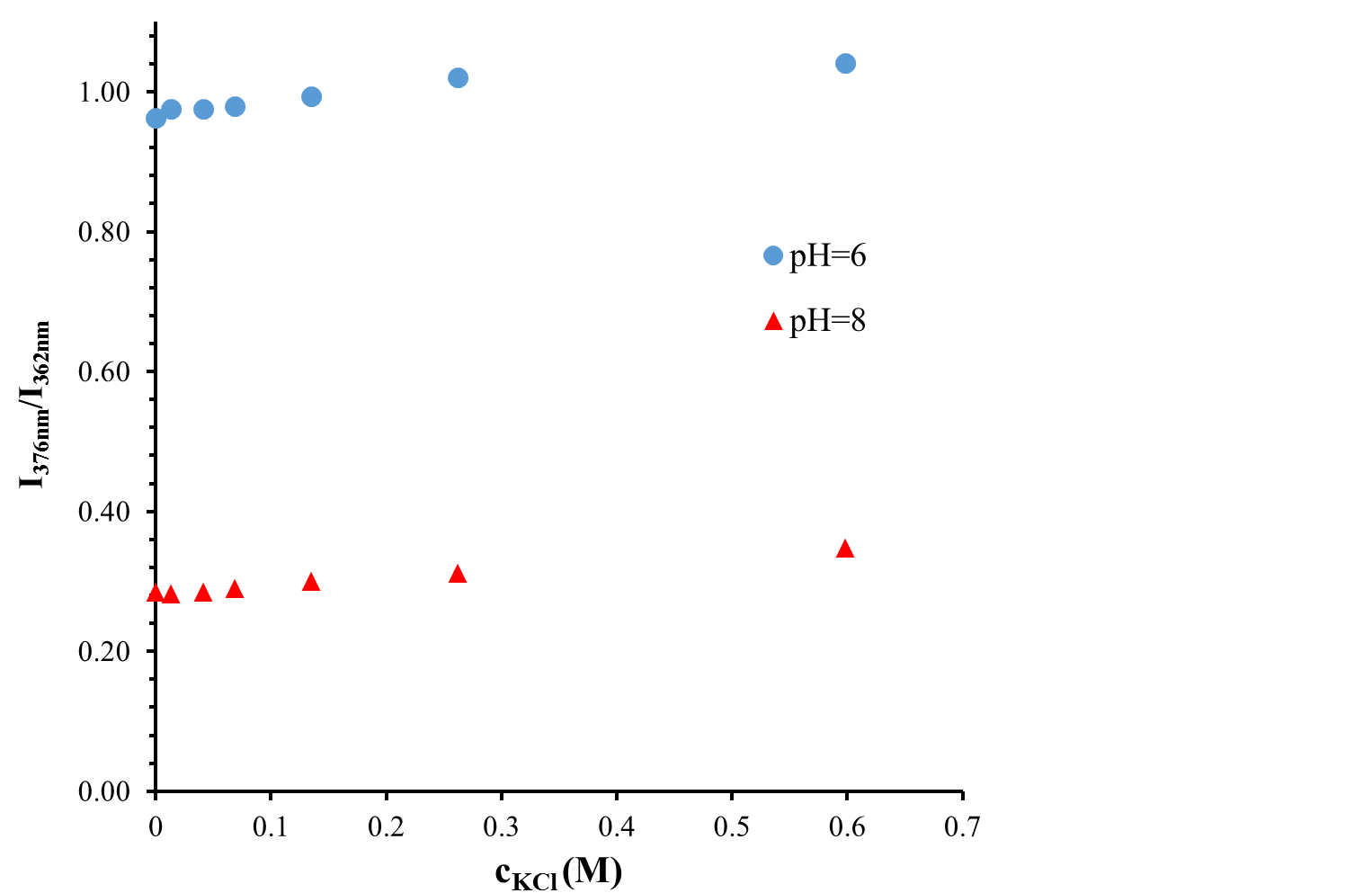


1. The effect of salt on the fluorescence of diMICAAc in BRB buffers at pH=6 and 8.
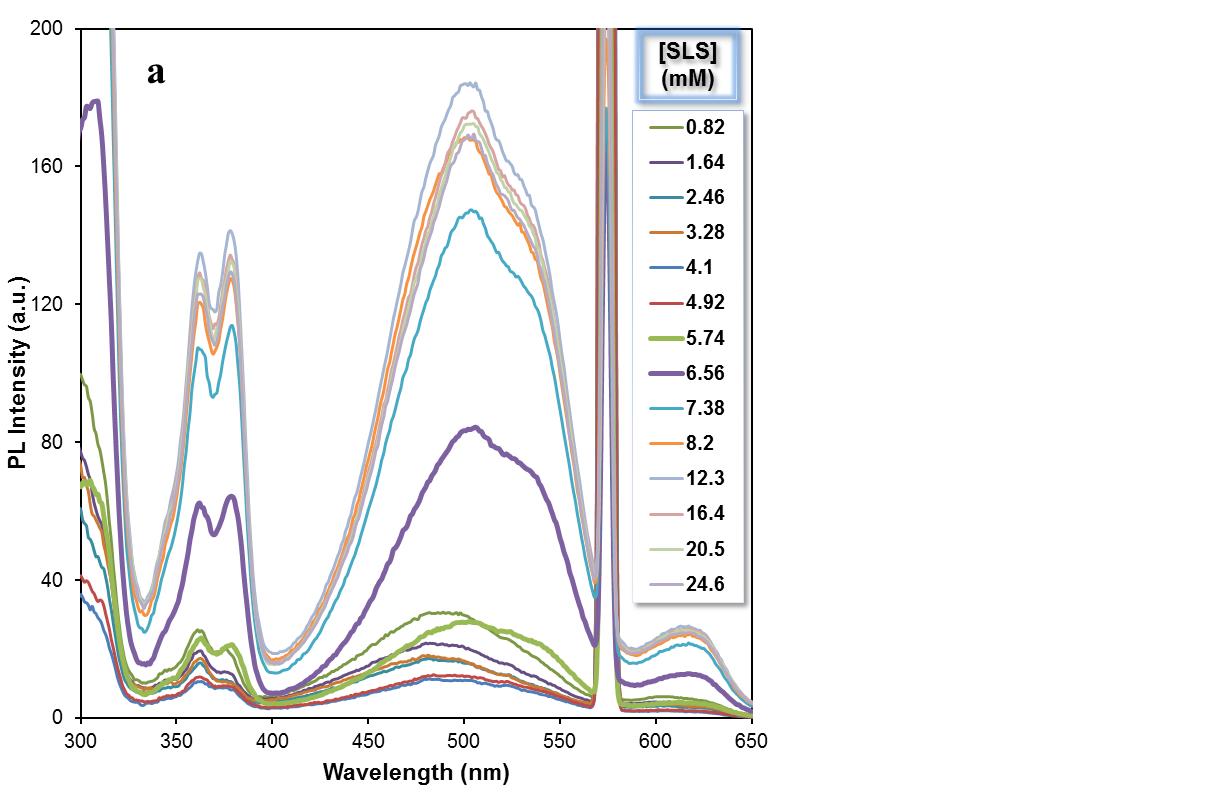

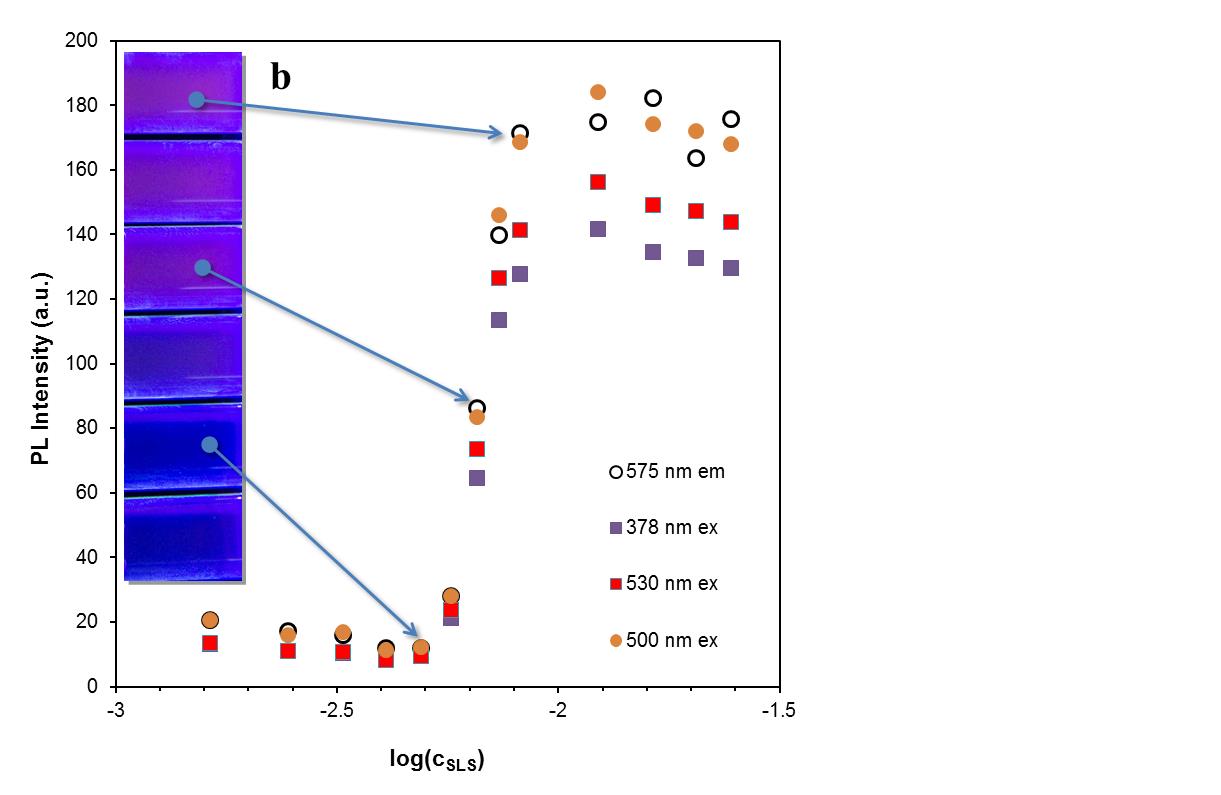

2. Fluorescent spectral changes of **diMICAAc** versus SLS concentration in water: (**a**) excitation spectra (**b**) PL Intensity maxima obtained from the emission (*em*) and from the excitation (*ex*) spectra *vs* SLS concentration. The inset picture shows the visible emission of SLS solutions near cmc containing **diMICAAc** illuminated by λ_ex_=365 nm UV-light. (T=20 ºC, [dye] = 4.58×10^-6^ M, V=3.00 cm^3^)


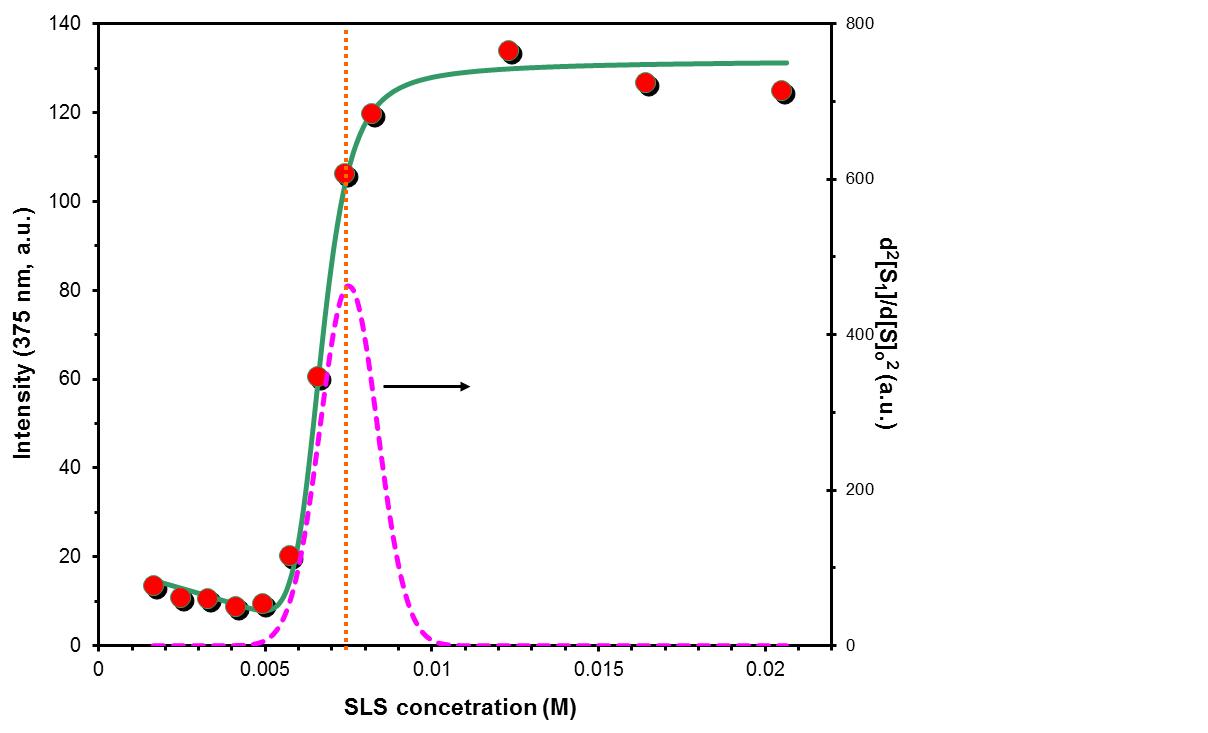


1. Variation of the intensity of the excitation peak at 375 nm as a function of SLS concentration

The dashed curve is obtained by the derivation of the fitted line

# Chapter V. Metal complexation studies


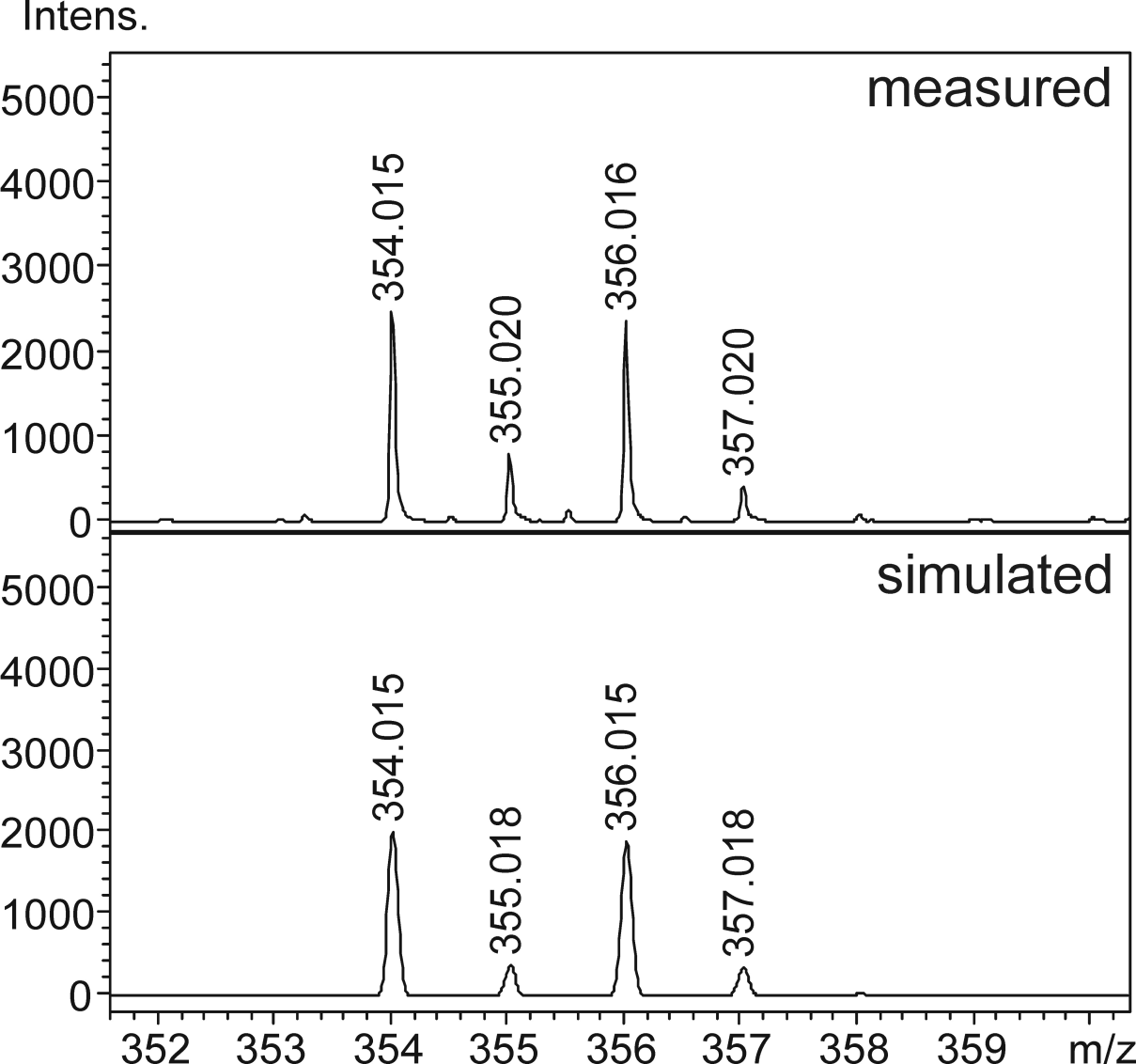


1. The measured and calculated ESI-MS spectra of Ag[(C_16_H_13_N_3_)]^+^ or Ag:diMICAAc 1:1 complex

1. The calculated emission spectra of diMICAAc (black line) and Ag:diMICAAc (dashed line) 1:1 complex in dioxane.

1. The calculated UV-vis absorption spectra of the two Ag:diMICAAc complexes together with the diMICAAc (grey line)

The ground state Gibbs free energy difference between the two forms is only 0.9 kcal mol^−1^

1. The calculated UV-vis absorption spectra of ICAAc in different solvents

1. The calculated emission spectra of ICAAc in different solvents.

1. The calculated UV-Vis absorption spectra of monoMICAAc in different solvents

1. The calculated emission spectra of monoMICAAc in different solvents

1. The calculated UV-Vis absorption spectra of diMICAAc in different solvents

1. The calculated emission spectra of diMICAAc in different solvents.

1. Emission spectra of the truncated ICAAc molecule (dashed lines) together with the emission (grey line) and UV-Vis absorption (orange line) of the original ICAAc

1. Relaxed PES scan of the N-H distance (protonation of the amino group) in the S1 state

1. Relaxed PES scan of the O-H distance (deprotonation of the amino group) in the S1 state

Computational Details

All calculations were performed using the Gaussian09 E.01 software package.^[1]^ We employed DFT and TDDFT for geometry optimizations at the ground and excited states respectively. The chosen functional was the M06^[2]^ with the TZVP basis set^[3]^ which method proved to be very successful in our previous works.^[4]^ On the silver atom we used the CRENBL ECP/basis.^[5]^ The calculations were done using the IEF-PCM solvent model.^[6-9]^

1. M. J. Frisch, G. W. Trucks, H. B. Schlegel, G. E. Scuseria, M. A. Robb, J. R. Cheeseman, Gaussian 09, revision E.01, Gaussian Inc., Wallingford CT, USA, **2009**.
2. Y. Zhao, D. G. Truhlar, *Theor. Chem. Acc.* **2008**, *120*, 215.
3. A. Schaefer, C. Huber, R. J. Ahlrichs, *Chem. Phys.* **1994**, *100*, 5829.
4. M. Nagy, D. Rácz, Z. L. Nagy, T. Nagy, P. P. Fehér, M. Purgel, M. Zsuga, S. Kéki, *Dyes and Pigments* **2016**, *133*, 445.
5. L. A. LaJohn, P.A. Christiansen, R. B. Ross, T. Atashroo, W. C. Ermler, *J. Chem. Phys.* **1987**, *87*, 2812.
6. J. Tomasi, B. Mennucci, R. Cammi, *Chem. Rev.* **2005**, *105*, 2999.
7. G. Scalmani, M. J. Frisch, *J. Chem. Phys.* **2010**, *11*, 114110.
8. J. Tomasi, B. Mennucci, E. Cances, *THEOCHEM* **1999**, *464*, 211.
9. J. L. Pascual-Ahuir, E. Silla, I. Tunon, *J. Comput. Chem.* **1994**, *15*, 1127.

# Chapter VII. Biological studies

***Overview of the staining capacity of the new dyes for fluorescence microscopy and cytometry in live human cells.***

To compare the applicability of the dyes for live cell imaging a human cervix carcinoma cell line HeLa cells cultured in ibidi 8-well microscopy chambers were stained with 0.3 μg/ml dye for 30 minutes in dark applying cell-culture conditions. The results are presented in Figure S36a as a 4x3 mosaic. In the first column, bright field images show HeLa cells (A/1, B/1, C/1), where **diMICAAc** stained cells demonstrated the best-preserved morphology (C/1). Column #2 images indicate that the UV-wavelength range illumination provides the most intense excitation for all dyes. Column #3 and #4 images show that **ICAAc** and **monoMICAAc** are not applicable with higher wavelength filter sets. **diMICAAc**, however, has visually feasible signal to noise ratio with the blue/green and green/red filter sets, as well. Viewing the fine pattern of the cell staining in column #2, **ICAAc** probably binds cell membranes more, since it resolved the cytoplasmic region and the whole cell body. However, it is excluded from the cell nucleus; consequently, it probably binds DNA less (A/2). The opposite is valid for **monoMICAAc**, since it stained cell nuclear structures, probably the DNA (B/2). **DiMICAAc** probably binds cell membranes, some unknown intracellular vesicular structures, probably the DNA slightly, and possible binds to some extent to the RNA, since in many cells, above the nucleus, nucleolar structures could be resolved as well.

| **a** 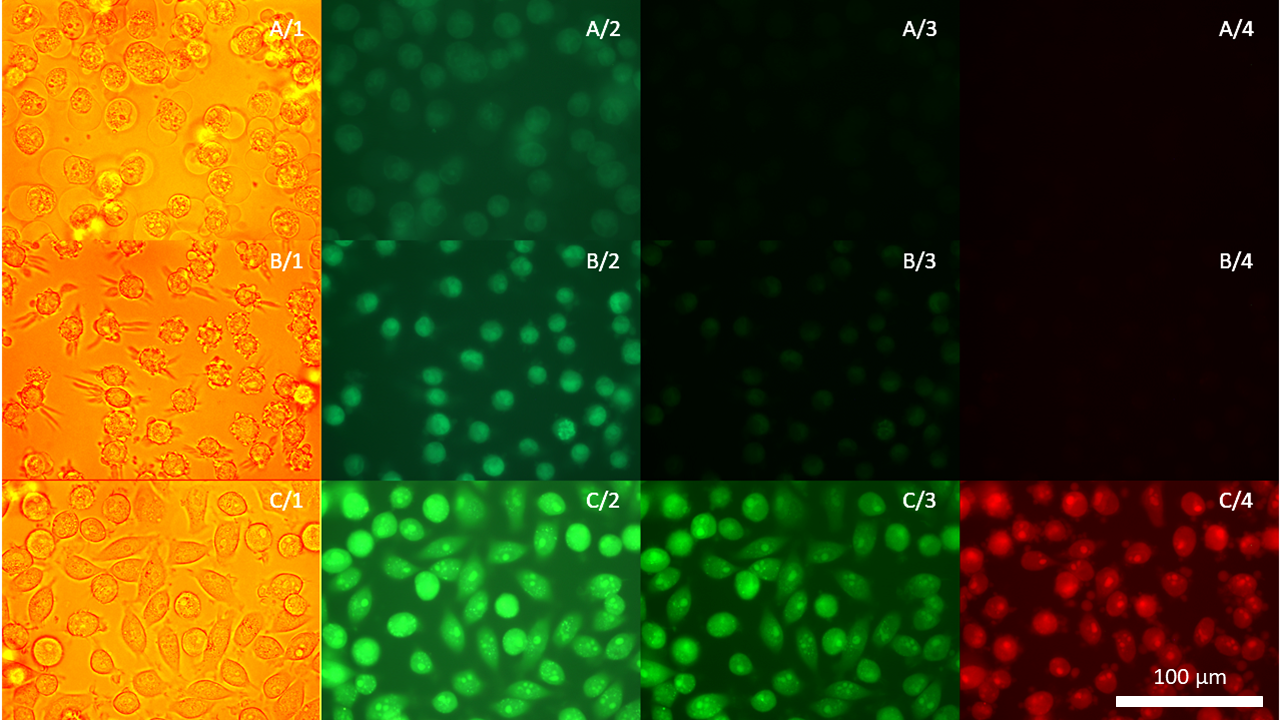 |
| --- |
| **b**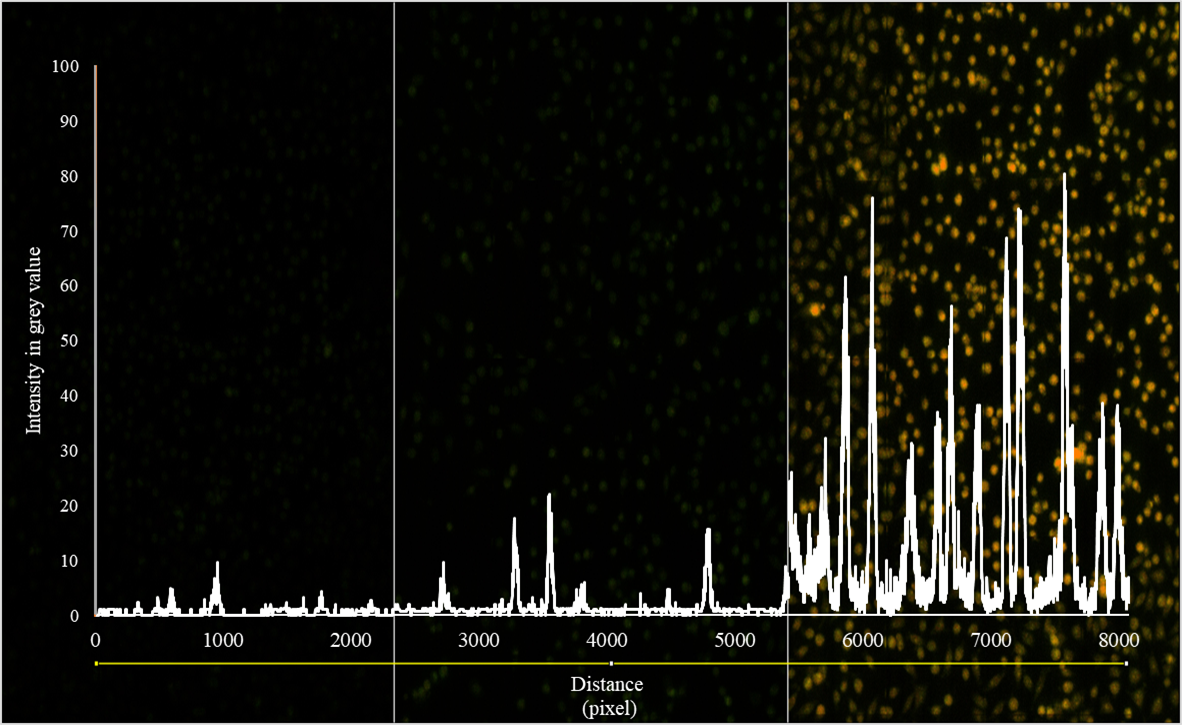 |

**Figure S36** (**a**) Demonstration of the staining capacity of **ICAAc** derivatives for live cell imaging in HeLa cells. The following staining arrangement was applied: (A) **ICAAc**; (B) **monoMICAAc**; (C) **diMICAAc**. In column #1: bright field; in column #2: UV/blue fluorescence; in column #3: blue/green fluorescence; in column #4: green/red excitation/emission epifluorescence illumination was employed. (**b**) Quantitative comparison of cell staining capacity of the new dyes applying imaging cytometry. Mosaic scan images of vitally stained HeLa cells were made for the **ICAAc** (left panel), **monoMICAAc** (middle panel), and **diMICAAc** dyes (right panel) applying iCys laser-scanning cytometer with the same excitation and detection settings. 488 nm laser illumination and 530±15 nm emission were used to make images. The cell staining intensity profile was plotted on the line indicated by the yellow line below the x-axis.

All three dyes are applicable for 488 nm laser illuminated live cell fluorescence imaging at 0.6 μg/ml dye concentration, as it is seen in FigS37 A/1-2-B/1-2-C/1-2. However, **diMICAAc**, which has the best optical property and live cell staining capacity, can be used at a lower 0.3 μg/ml dye concentration, as well (FigS37 C/3-4). Dyes abruptly stain live cells at 37 °C, since there was no noticeable difference between the fluorescence intensity of cells within an hour of immediate examinations in a heated stage incubator of a microscope (FigS37 C/3-4). Our initial experience with these dyes was that they are quite nontoxic (LD_50_ values were 7.27, 5.78, and 7.50 μM for **ICAAc**, **monoMICAAc** and **diMICAAc**, respectively) in acute experiments, since in short-term cytometric examinations, where a vital fluorescent cell death indicator propidium iodide was also included in the medium, for one-three hours, HeLa cells preserved their full viability and only very few cells turned red, indicating the cell membrane leakage and cell death (FigS37 A/3-4, B/3-4, C/3-4).

In live cell imaging, as it is visualized in Figure S36b, the highest fluorescent signal at the 488 nm laser line could be obtained. Furthermore, we quantified the live cell staining capacities of the three dyes in HeLa cells applying imaging cytometric measurements. We have determined the following signal to noise ratio values 3.95, 8.01, and 9.37 for the **ICAAc**, **monoMICAAc**, and **diMICAAc** dyes, respectively, using the 488 nm laser light excitation and 530/30 nm emission detection. The 405, 567 and 633 nm laser lines are not so feasible to use with these dyes (data not shown).


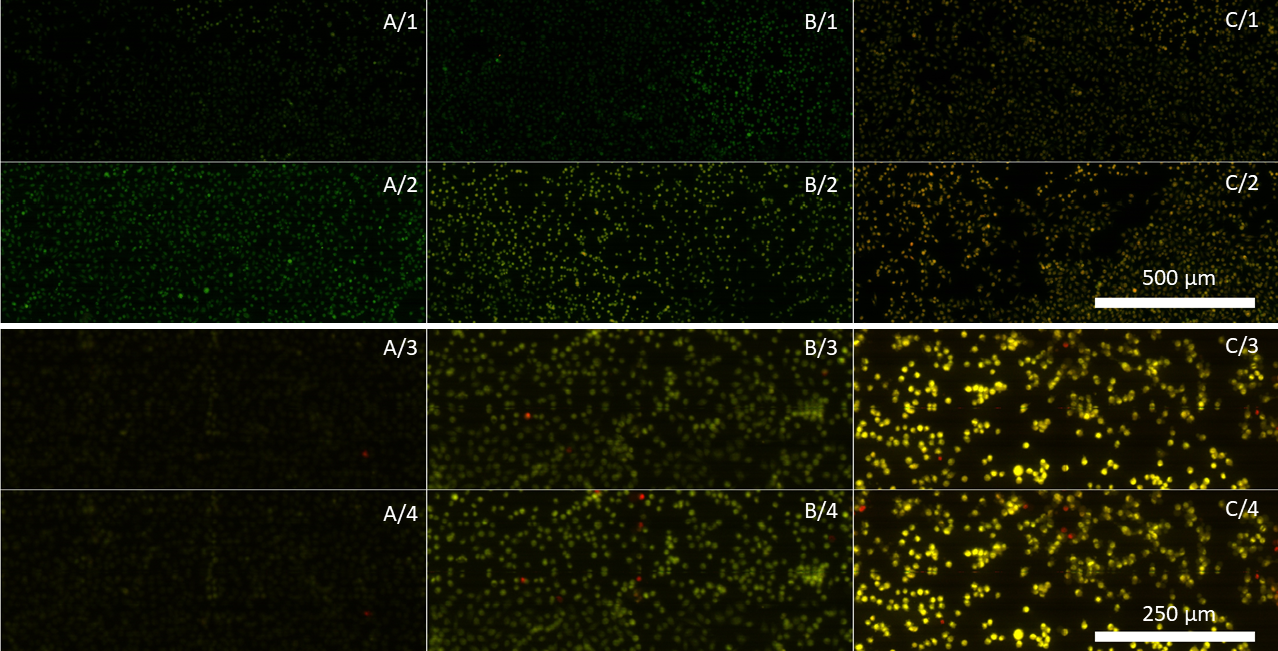


1. Montage of well images captured by an iCys imaging cytometer to evaluate fluorescence staining and acute toxicity of the three dyes

Well images are build up from 4x4 field images (each is a 1024x768 pixel of 0.25 μm) in the upper six panels (A/1-C/2) and in the lower six panels (A/3-C/4) it is from 2x2 field images (with the same pixel size; the border of the image stitching is unnoticeable). The tiny bright spots are single cells in all images, where the greenish-yellowish color represents dye staining from the three different compounds (column A: ICAAc; column B: monoMICAAc; and column C: diMICAAc) and the red color is from the vital iodide dye indicating dead cells (just very few dead cells is visible).

In the 1^st^ row images, 0.3, and in the 2^nd^ row images, 0.6 μg/ml dye concentration was used to stain live cells for 30 minutes; cytometric measurements were made with different photomultiplier voltages (in the green channel 38V, 28V, and 22V for A/1-2, B/1-2, and C/1-2, respectively) to optimize images for the different dye intensities.
In the bottom two row images, 0.3 μg/ml dye concentration was applied for an immediate observation, shown in the 3^rd^ row; and for an observation with an hour later, shown in the 4^th^ row images; green channel photomultiplier voltages were set in all A/3-4, B/3-4 and C/3-4 images to the same 20V. This helped to compare the staining capacity difference for the dyes. Samples of all images were illuminated by the 488 nm laser light and fluorescence emission was detected with a 530/30-nm band-pass (green channel) and a 650 nm longpass (red channel) filter set with a 40x LWD (NA 0.6) objective.

### Cell line

Human cervix epithelioid carcinoma HeLa cell line was obtained from Sigma Aldrich, Hungary. Cells were cultured in Dulbecco’s Modified Eagle’s Medium (DMEM) containing 10 % heat-inactivated fetal bovine serum, 2 mM L-glutamine, 100 units/ml penicillin and 100 μg/ml streptomycin applying 5 % CO_2_ and 100 % humidity. Every second day, cultures at ~85 % confluency were routinely split 1:5 in T25 tissue culture flasks. The day before an imaging experiment, cells were subcultured into an ibidi 8-well microscopy chamber at 25000 cells per well cell concentration.

### Laser-Scanning Cytometry and microscopy

Slide-based laser-scanning cytometry (LSC), conventional bright field and epifluorescence microscopy were used for live-cell imaging with parallel detection of cell death. The imaging system used was an iCys Research Imaging Cytometer (Thorlabs Imaging Systems, Sterling, VA; formerly CompuCyte) equipped with a DP71 high-resolution digital color camera (Olympus, Olympus Hungary Kft. Budapest) and an ibidi stage incubator (ibidi, Zenon Kft. Hungary, Szeged). The extended iCys system is based on an Olympus IX-71 inverted microscope equipped with four lasers, four photomultipliers for fluorescence detection, and two photodiodes for chromatic absorbance detection. The system is controlled by iCys 7.0 software, iNovator Application Development Toolkit software (Thorlabs) and Cell^B camera software (Olympus). Scanning of the samples by the laser beam took place pixel by pixel applying a fixed offset from the bottom of the coverslip to place the focus to the middle plane of the cells. User-defined areas in the specimen with optimal cell density were marked as regions of interest (4x4 or 2x2 ROIs) and scanned in an automated manner repeatedly. For live cell imaging, ibidi stage incubator was set to 37 °C temperature applying 5 % CO_2_ and 90 % humidity. All fluorescent dyes were used in 0.3 and 0.6 μg/ml final concentration, excited with 488 nm solid-state laser, and fluorescence emission was detected with 530/30 and 580/30 nm band-pass and 650 nm longpass filters. The light source of the epifluorescence microscope was a 100 W mercury lamp, and we applied regular UV/blue, blue/green, and green/red excitation/emission filter sets (Olympus). The following fluorescence filter cubes were used. For UV/blue imaging the U-MWU filter cube with 330-385 nm excitation filter, 400 nm dichroic mirror and 420 nm emission filter; for blue/green imaging the U-MWB filter cube with 450-480 nm excitation filter, 500 nm dichroic mirror and 515 nm emission filter; and for the green/red imaging the U-MWG filter cube with 510-550 nm excitation filter, 570 nm dichroic mirror and 590 nm emission filter. For acute cell toxicity measurements, in some cases, the propidium iodide vital stain was also added to the medium in 10 μg/ml final concentration.

### MTT cell viability assay

For dye toxicity tests the human cervix carcinoma HeLa cell line was used. After three days of the cell splitting, cells were trypsinized, collected and counted into single cell suspension of fresh culture medium. Aliquots (200 μL) of each cell suspension were placed in wells of 96-well-plates (Biocenter, Szeged, Hungary). Cell number was set to 12,000 cells per well. Six different concentrations of each derivative were tested in triplicates of samples. The derivatives were dissolved in DMSO (1 v/v%) in 1 mg/ml concentration and an appropriate amount of the stock solution and DMEM culture medium was added to the wells to produce the required dye concentration. Cells were incubated for 24 hours in culture conditions and for the last 2 hours, half of the culture medium was replaced by 100 µl of MTT (Sigma Aldrich, Budapest, Hungary) solution (0.5 mg/ml in PBS). Then, the culture medium was removed and the MTT formazan was extracted with 100 µl of DMSO applying gentle agitation on a shaker. After 10 minutes at room temperature, absorbances were read at 570 nm using an automatic plate reader (Synergy HT reader, Bio-Tek Instruments, Winooski, VT, USA). Percentage viability of the population of cells in each well was calculated according to the following equation: (Absorbance of treated cells – Blank) / (Absorbance of control cells - Blank) × 100.


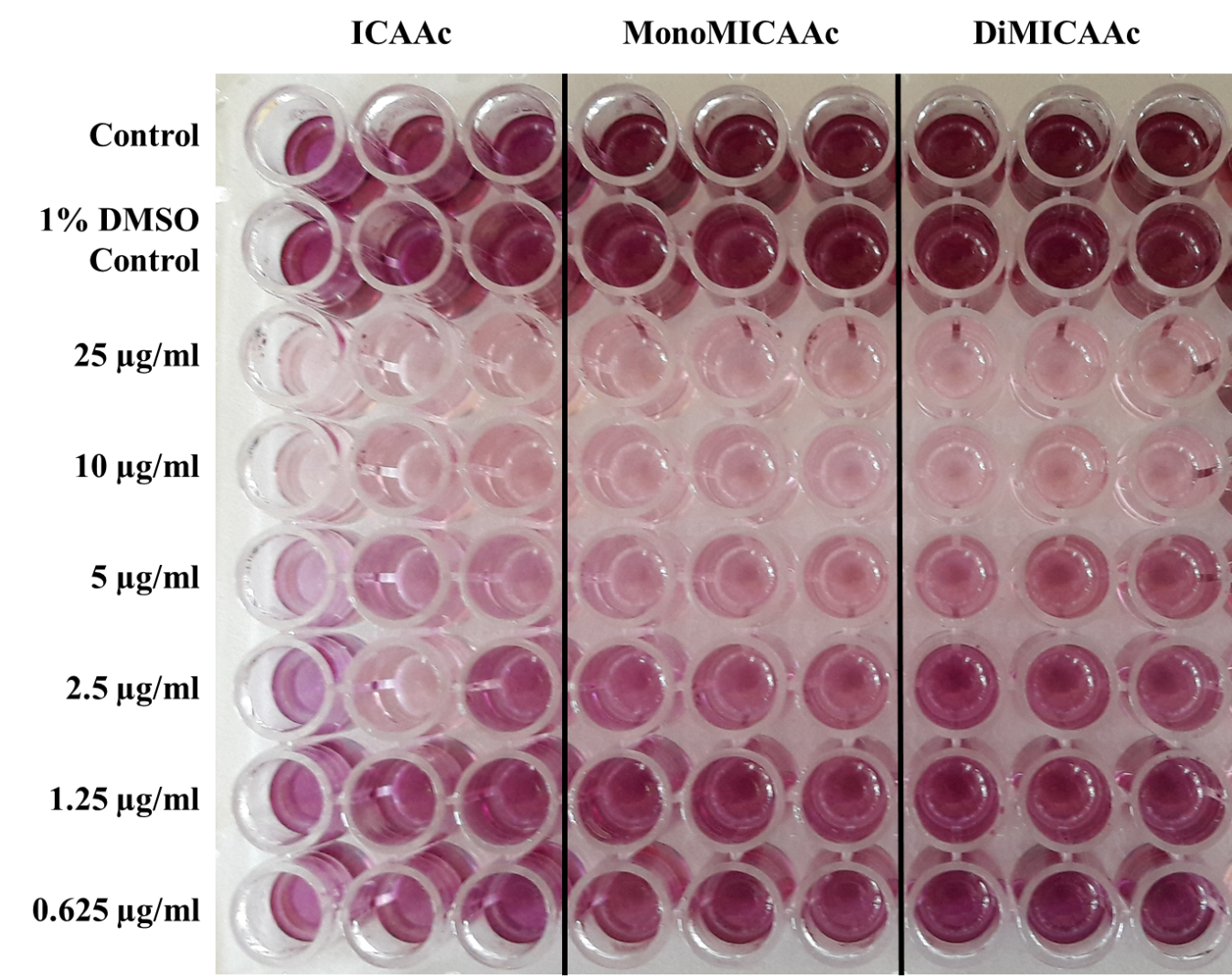


1. Results of the dye toxicity test employing the colorimetric cell viability MTT test in a 96-well plate on HeLa cells

1. Viability curve of the ICAAc

1. Viability curve of the monoMICAAc

1. Viability curve of the diMICAAc
